# Integrated Radiobiotherapy: A State-Based Framework for Response-Adaptive Combination Oncology

**DOI:** 10.3390/curroncol33100637

**Published:** 2026-10-20

**Authors:** Srinivasan Vijayakumar, Marc Matrana, Vani Vijayakumar

**Affiliations:** 1 Department of Radiology, Ochsner Health System, New Orleans, LA 70121, USA; vani.vijayakumar@ochsner.org; 2 Department of Radiation Oncology, Kasturba Medical College, Manipal Academy of Higher Education, Manipal, India; 3 Ochsner MD Anderson Cancer Center, New Orleans, LA 70121, USA; mamatrana@ochsner.org

**Keywords:** integrated radiobiotherapy, state-based framework, SABR, SBRT, spatially fractionated radiation therapy, targeted radionuclide therapy, response-adaptive oncology

## Abstract

**Background:** Cancer care increasingly requires coordinated use of systemic therapy, targeted radionuclide therapy (TRT), and external-beam radiotherapy (EBRT), yet clinical workflows often remain organized around static staging and sequential specialty handoffs. **Approach:** We propose Integrated Radiobiotherapy (IRBT), a state-based, response-adaptive research framework in which molecular imaging, systemic therapy, TRT, and EBRT can be evaluated as coordinated components of prospective studies. The framework uses shared selection criteria, response measures, and prespecified adaptation options—escalate, consolidate, switch, or de-escalate. The oligometastatic and oligoprogressive spectrum serves as a pragmatic clinical testbed, while two landmark 1995 papers provide historical context for the implementation discipline required. **Key Recommendations:** Trials should evaluate sequencing at clinically relevant decision points, use state-based endpoints that capture lesion-level control and spatial heterogeneity, establish minimum governance standards with multidisciplinary peer review and cumulative-toxicity safeguards, and address equity, ethics, and resource intensity as core implementation requirements. **Conclusions:** Although individual components—including oligometastatic stereotactic body radiotherapy, TRT, and adaptive EBRT—have prospective support, the fully integrated response-adaptive strategy remains unvalidated. IRBT offers a proposed structure and research agenda for multimodality oncology trials; it is not a clinical practice guideline or treatment mandate. *“Those who cannot remember the past are condemned to repeat it.”*—George Santayana.

## Simple Summary

Cancer care is increasingly defined by dynamic disease states, yet radiation workflows remain organized around static staging and specialty handoffs. Integrated radiobiotherapy proposes a state-based architecture with shared selection criteria, endpoints, and explicit adaptation rules: escalate, consolidate, switch, and de-escalate. Individual components (oligometastatic SBRT, TRT, and adaptive EBRT) have prospective support; the fully integrated approach requires validation—this is a research and program-design agenda, not a practice mandate. Implementation priorities include sequencing trials at real decision points, state-based endpoints, minimum viable governance, cumulative toxicity guardrails, and disease-specific protocols. Equitable access, ethical informed consent, and value demonstration are essential to responsible implementation. Modern cancer care is moving beyond static anatomical staging toward dynamic biological adaptation, while radiation-delivery workflows often remain organized around fixed treatment schedules. This conceptual paper presents Integrated Radiobiotherapy (IRBT), a state-based, response-adaptive research framework in which stereotactic body or ablative radiotherapy, spatially fractionated radiotherapy, targeted radionuclide therapy, and systemic biotherapies can be evaluated as coordinated components of prospective studies. The framework organizes investigation around target expression, spatial heterogeneity, systemic tumor burden, and organ-at-risk safety constraints and proposes adaptation options such as escalation, consolidation, switching, or de-escalation. IRBT is an educational roadmap and hypothesis-generating research agenda, not a validated clinical decision system or practice mandate.

## 1. Introduction

Cancer management is increasingly defined by therapeutic change over time: target volumes evolve, metastatic lesions diverge biologically across organ sites, and modern systemic agents convert rapidly fatal disease into extended sequences of response, acquired resistance, and repeat intervention. Yet radiation oncology workflows remain constrained by static baseline staging and sequential specialty handoffs. Integrated Radiobiotherapy (IRBT) is proposed as a state-based, response-adaptive research framework for examining coordinated use of molecular imaging, targeted systemic therapy, targeted radionuclide therapy (TRT), and external-beam radiotherapy (EBRT). The framework specifies shared selection criteria, response measures, and adaptation rules so that therapeutic sequencing can be evaluated prospectively and, if validated, developed into reproducible multidisciplinary research programs.

### Terminology & Core Definitions

To ensure clarity across international treatment guidelines and emerging paradigms in radiation science, we use four core terms. First, stereotactic body radiation therapy (SBRT) refers to the precise, image-guided delivery platform used for extracranial stereotactic treatment, whereas stereotactic ablative radiotherapy (SABR) emphasizes ablative intent and commonly denotes high-dose hypofractionated regimens, often with BED_10_ greater than 100 Gy, delivered for definitive local control [1,2,3]. Because usage varies across the literature, this manuscript uses SBRT for the delivery technique and SABR when emphasizing ablative intent. Second, radiobiotherapy is the deliberate, biologically synchronized integration of radiation therapy with modern systemic biotherapies, including molecularly targeted agents and immune checkpoint inhibitors, and is distinct from historical concurrent chemoradiation with cytotoxic agents [4,5]. Third, Integrated Radiobiotherapy (IRBT) is a proposed research architecture using shared patient-selection criteria, standardized response metrics, and prespecified adaptation rules that can be evaluated prospectively in clinical trials [4,5]. Fourth, radiobiomedicine is the overarching academic and clinical discipline encompassing biology-guided physical radiation across biological systems and extending beyond oncology to non-malignant, inflammatory, and neurofunctional indications [4,6,7]. Historically, orthovoltage radiation was used for benign and inflammatory conditions—a paradigm advanced by pioneers such as Sarah Donaldson at Stanford—but these applications lacked contemporary molecular and radiobiological underpinnings [8,9,10]. Modern radiobiomedicine revisits this field by pairing organ-sparing, submillimeter precision with targeted cytokine, macrophage, and neuromodulatory biology [6,11]. This expansion is reflected in professional initiatives such as the American Society for Radiation Oncology Functional Radiation Medicine Task Force, which highlights low-dose radiation therapy for anti-inflammatory, tissue-sparing control in osteoarthritis and radiosurgical capsulotomy for refractory psychiatric disorders [6,12]. Radiobiomedicine therefore serves as the master discipline—parallel to the historical expansion of interventional radiology—under which Integrated Radiobiotherapy, addressing oncologic multi-agent coordination [5,13], and Functional Radiation Medicine, addressing non-oncologic tissue-sparing applications [6,11,12], reside. Table 1 expands on the definitions of the terminologies being proposed.

The integrated, response-adaptive strategy described in this manuscript is intended solely as an educational construct and hypothesis-generating framework. Although individual components—such as focal stereotactic ablative radiotherapy, multisite SABR, targeted radionuclide therapy, and adaptive EBRT—have supporting clinical or technical evidence, the fully coordinated, biologically integrated approach proposed here has not yet been validated in prospective randomized trials [1,14,15,16,17,18]. Whether multisite biological integration improves overall survival, progression-free survival, symptom control, or quality of life compared with standard sequential therapies remains unknown. Rigorous prospective studies are required to determine clinical benefit, safety, and cost-effectiveness and to assess whether the added complexity and resource utilization are justified. Supplementary File S4 provides a draft international prospective registry protocol to support standardized real-world evaluation and future validation of the IRBT framework. Until such data mature, this framework should be interpreted strictly as an educational model, conceptual roadmap, and research-design tool—not as a clinical practice guideline, treatment recommendation, or standard of care [5].

## 2. From Stage-Based to State-Based Radiation Medicine

Traditional clinical oncology relies heavily on anatomical staging systems such as the AJCC TNM framework to categorize disease extent and determine initial management strategies [19]. Although anatomical staging remains essential for population-level prognostication, it lacks the temporal and biological granularity required to guide modern response-adaptive therapy [20,21]. In contemporary practice, patients presenting with identical stage IV diagnoses may exhibit different clinical trajectories depending on driver-mutation status, PD-L1 expression, circulating-tumor-DNA dynamics, and microenvironmental heterogeneity [21,22,23]. To address these limitations, the Integrated Radiobiotherapy (IRBT) framework shifts the primary organizing logic from static anatomical stages to dynamic disease states [20,21,22]. A clinical state is defined as an actionable biological and anatomical window characterized by four interacting variables: (1) Target Expression and Molecular Imaging Phenotype: The density, distribution, and functional activity of therapeutic targets measured through molecular imaging, such as PSMA, FDG, and somatostatin receptor PET, or tissue-based biomarkers [24,25]. (2) Spatial Heterogeneity and Lesion Geometry: The physical distribution of disease, including focal versus bulky disease and hypoxic cores versus vascularized peripheries, which informs consideration of uniform ablative SBRT/SABR or spatially fractionated radiation therapy [26]. (3) Systemic Tumor Burden and Tempo: Lesion number, disease distribution, and rate of progression over time, which help distinguish oligometastatic, oligoprogressive, and polymetastatic states and inform treatment intent [1,22,27]. (4) Organ-at-Risk and Hematologic Constraints: Normal-tissue tolerance, prior radiation exposure, and baseline hematologic or immune status, including absolute lymphocyte count, establish safety boundaries for protocol-based treatment planning [28,29].

### 2.1. Adaptive Reassessment and Treatment Selection

By continuously re-evaluating these four variables at prespecified treatment nodes, clinicians can systematically adapt radiation delivery within prospective protocols—for example, by escalating dose to focal resistant sites, limiting irradiated volumes to reduce normal-tissue and hematologic exposure, or switching to targeted radionuclide therapy when systemic burden increases or other protocol-defined criteria are met [15,16,28,29].

### 2.2. Implications for Medical Oncology

**Figure 1:**
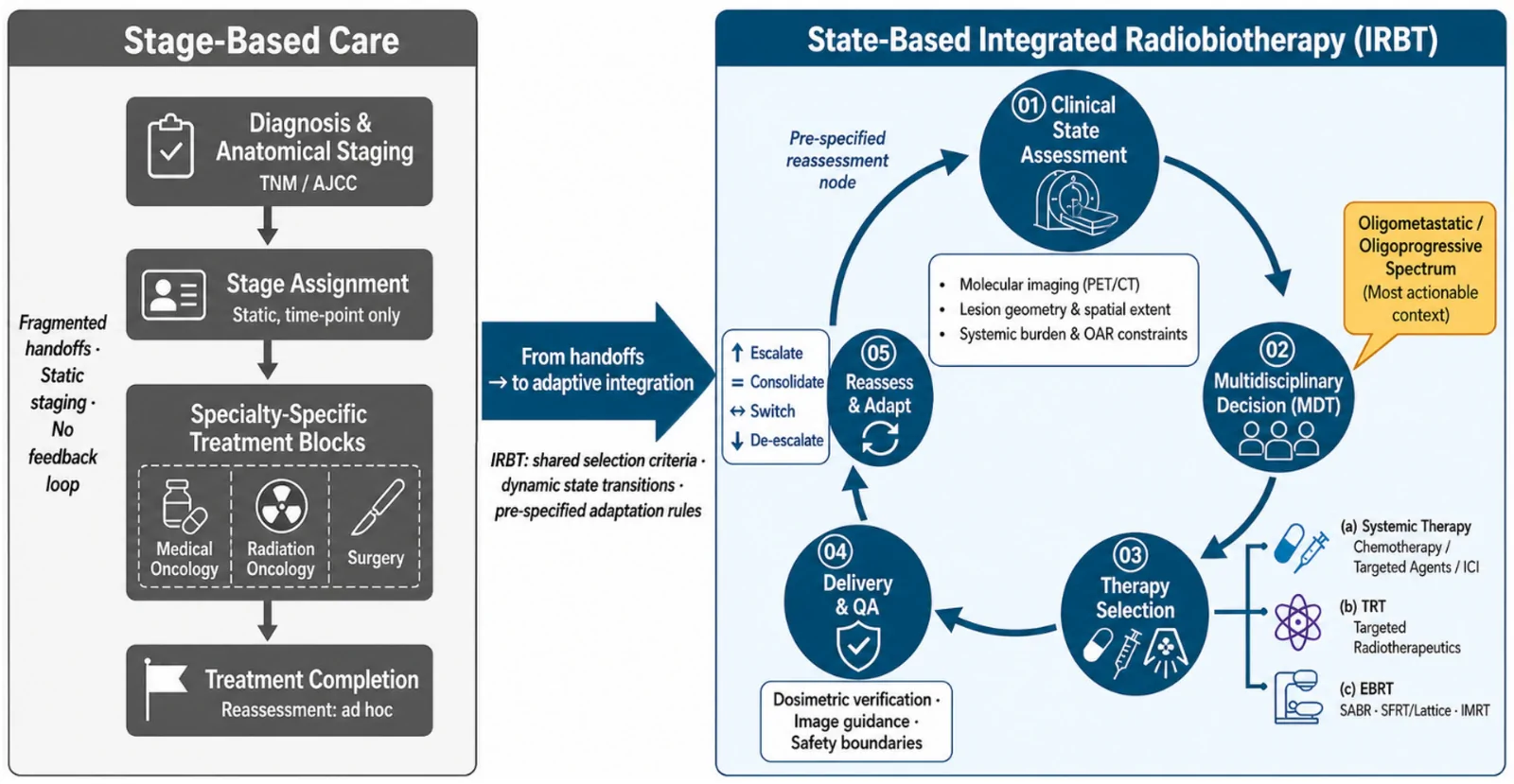
Comparison of conventional stage-based assessment with the proposed IRBT research framework. In the proposed model, clinical-state assessment informs multidisciplinary selection of systemic therapy, targeted radionuclide therapy, or external-beam radiotherapy, followed by protocol-defined delivery, reassessment, and possible adaptation. The figure presents a structure for prospective testing rather than a validated clinical decision system. Abbreviations: AJCC, American Joint Committee on Cancer; EBRT, external-beam radiotherapy; IRBT, Integrated Radiobiotherapy; MDT, multidisciplinary team; OAR, organ at risk; SABR, stereotactic ablative radiotherapy; SFRT, spatially fractionated radiotherapy; TNM, tumor–node–metastasis; TRT, targeted radionuclide therapy.

**Figure 2:**
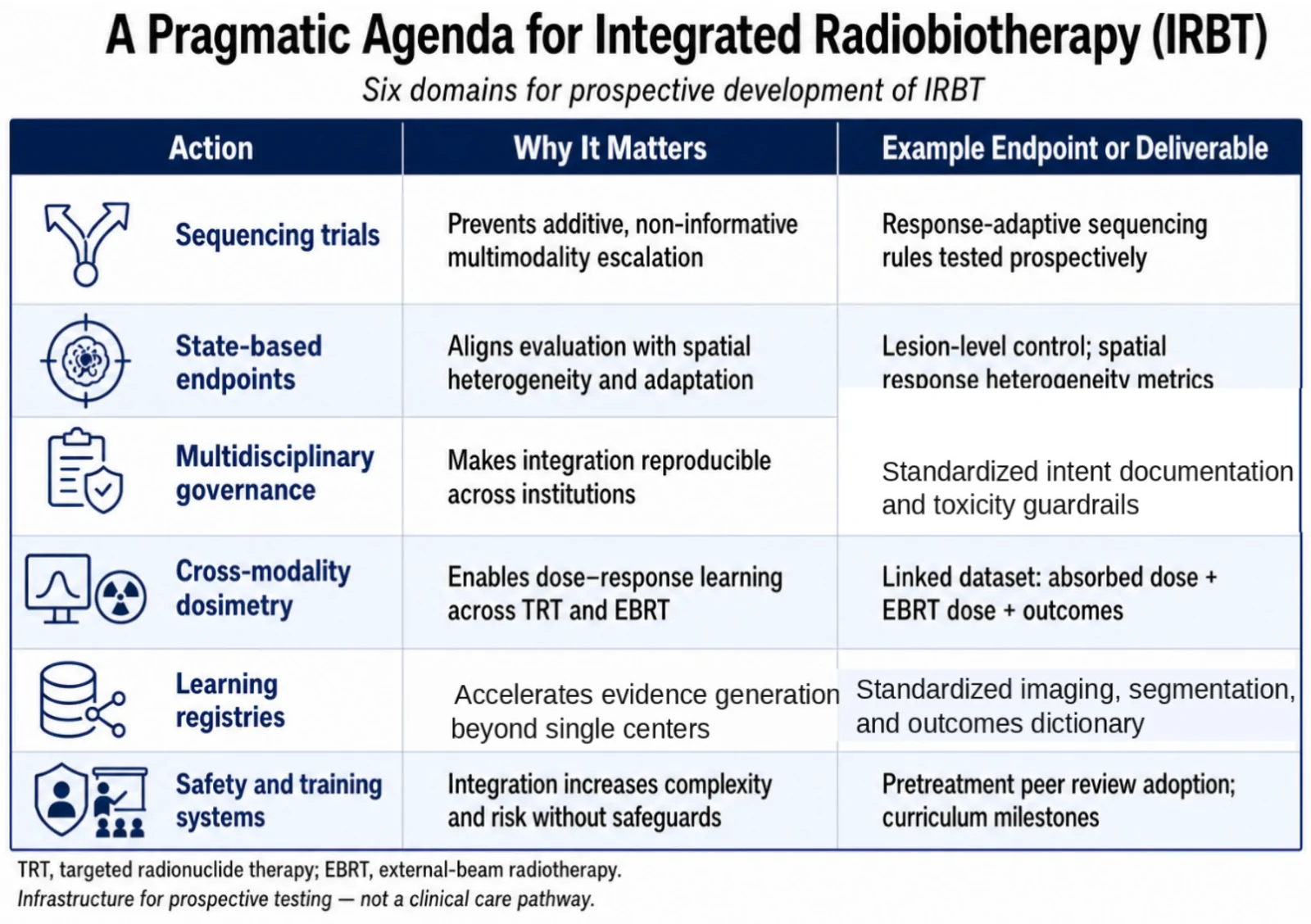
Research framework for prospective development of IRBT. The six domains are sequencing trials, state-based endpoints, multidisciplinary governance, cross-modality dosimetry, learning registries, and safety and training systems. The figure summarizes the infrastructure needed to test the framework reproducibly; it does not prescribe clinical care. Abbreviations: EBRT, external-beam radiotherapy; IRBT, Integrated Radiobiotherapy; TRT, targeted radionuclide therapy.

A state-based architecture reframes when to involve radiation and theranostics as components of prospective multidisciplinary investigation: (1) evaluating metastasis-directed radiotherapy for oligoprogression while an otherwise effective systemic regimen is continued or interrupted according to protocol [30,31,32,33,34,35]; (2) evaluating targeted radionuclide therapy when target expression and imaging support it, with lesion-level reassessment informing whether focal EBRT should be studied for residual or clinically dominant sites [15,25]; and (3) developing multidisciplinary sequencing plans with predefined reassessment timepoints and safety criteria [36,37,38]. Figure 1 and Figure 2 illustrate these principles. Figure 1 contrasts conventional stage-based assessment with a proposed IRBT research loop in which molecular imaging, systemic therapy, targeted radionuclide therapy, and external-beam radiotherapy are evaluated as coordinated inputs rather than parallel specialty tracks. Supplementary File S1 provides operational definitions of the clinical-state variables, the rationale for the five-step adaptive loop, and a glossary of harmonized terminology. Figure 2 translates the framework into six domains for prospective development: sequencing trials, state-based endpoints, multidisciplinary governance, cross-modality dosimetry, learning registries, and safety and training systems.

Figure 1 presents the comparison between conventional stage-based assessment and the proposed IRBT research framework.

## 3. Integrated Radiobiotherapy Modalities & Technical Platforms

To move from conceptual architecture to deployable clinical strategy, IRBT requires clear definition of the radiation and biologic platforms that serve as its operational building blocks in hypothesis generation and design of clinical trials.

### 3.1. Framework Modalities & Execution Foundations

To operationalize the state-based framework within interdisciplinary oncology, the Integrated Radiobiotherapy (IRBT) architecture uses distinct physical and biological delivery modalities and adaptive workflows to adjust plan geometry as spatial tumor volumes change [16]. Rather than treating external-beam radiotherapy and targeted radionuclide therapy (TRT) as isolated modalities, IRBT frames them as potentially complementary interventions selected according to molecular targets, matched diagnostic imaging, disease distribution, and treatment intent [15,25]. Translating these platforms into actionable decision structures requires a defined radiobiological rationale for therapeutic combinations [23,39], consideration of systemic immune effects from local radiotherapy [40,41], prospectively specified adaptive trial methods [36], and multidisciplinary coordination that reduces fragmented specialty handoffs [37,38]. These principles provide the foundation for the hypothesis-generating State-Based Framework Categorization (SBFC) system described below.

### 3.2. State-Based Framework Categorization (SBFC)

To convert complex, multivariable clinical presentations into structured trial decision nodes, we propose the hypothesis-generating State-Based Framework Categorization (SBFC) system. Its categories draw on established concepts in oligometastatic and oligoprogressive disease, local consolidative therapy, spatially fractionated radiotherapy, and cross-modality treatment integration [1,14,15,22,26,30,31,42,43].

SBFC-0 (Baseline Controlled Status): Disease exhibits uniform stability or complete response on systemic therapy. No state-based radiation intervention is proposed, and observation continues according to disease-specific standards. This category is an illustrative component of the proposed framework rather than a separately validated clinical state.

SBFC-1 (SBFC-OP—Oligoprogressive Transition): Emergence of a limited number of non-responsive focal lesions while systemic disease remains controlled elsewhere, creating an opportunity to evaluate local ablative therapy to control resistant subclones and prolong benefit from an otherwise effective systemic regimen [30,31,32,33,34,35].

SBFC-2 (SBFC-TPCI—Oligometastatic Presentation/Curative Intent): De novo low-volume metastatic presentation in which comprehensive treatment of all visible disease is evaluated with the aim of prolonging disease control [14,22,27,42,44,45].

SBFC-3 (SBFC-SyBP—Symptomatic Bulky Progression/Palliative Potential): Bulky, symptomatic, or organ-threatening progression may warrant specialized local interventions such as spatially fractionated radiotherapy (SFRT/GRID/Lattice) for symptom relief within appropriately designed protocols [26,43,46,47].

SBFC-4 (SBFC-AsyP—Asymptomatic High-Burden Progression/Palliative Potential): Rapid systemic progression characterized by high metabolic volume across multiple visceral organs without immediate local crises. In this proposed category, radiation would be limited to carefully selected palliative or sub-ablative approaches intended to minimize normal-tissue exposure and protect hematologic reserves [28,29].

SBFC-5 (SBFC-TINV—Investigational Cross-Modality Transition): Complex biological scenarios with active investigational options, such as combining systemic radioligand therapy with focal external-beam radiotherapy in prospective response-adaptive trials [15,48,49,50,51,52,53].

Supplementary File S2 provides an SBFC clinical worksheet that translates these proposed states into a structured research template for consistent trial classification, safety monitoring, and prespecified reassessment.

### 3.3. Hypothesis-Generating Nature of Clinical Thresholds

The SBFC categories and any associated thresholds are provisional constructs intended to support prospective trial design. They should not be interpreted as validated disease states, treatment indications, or assertions of curative potential.

## 4. Disease-Specific Applications & Clinical Scenarios

**Figure 3:**
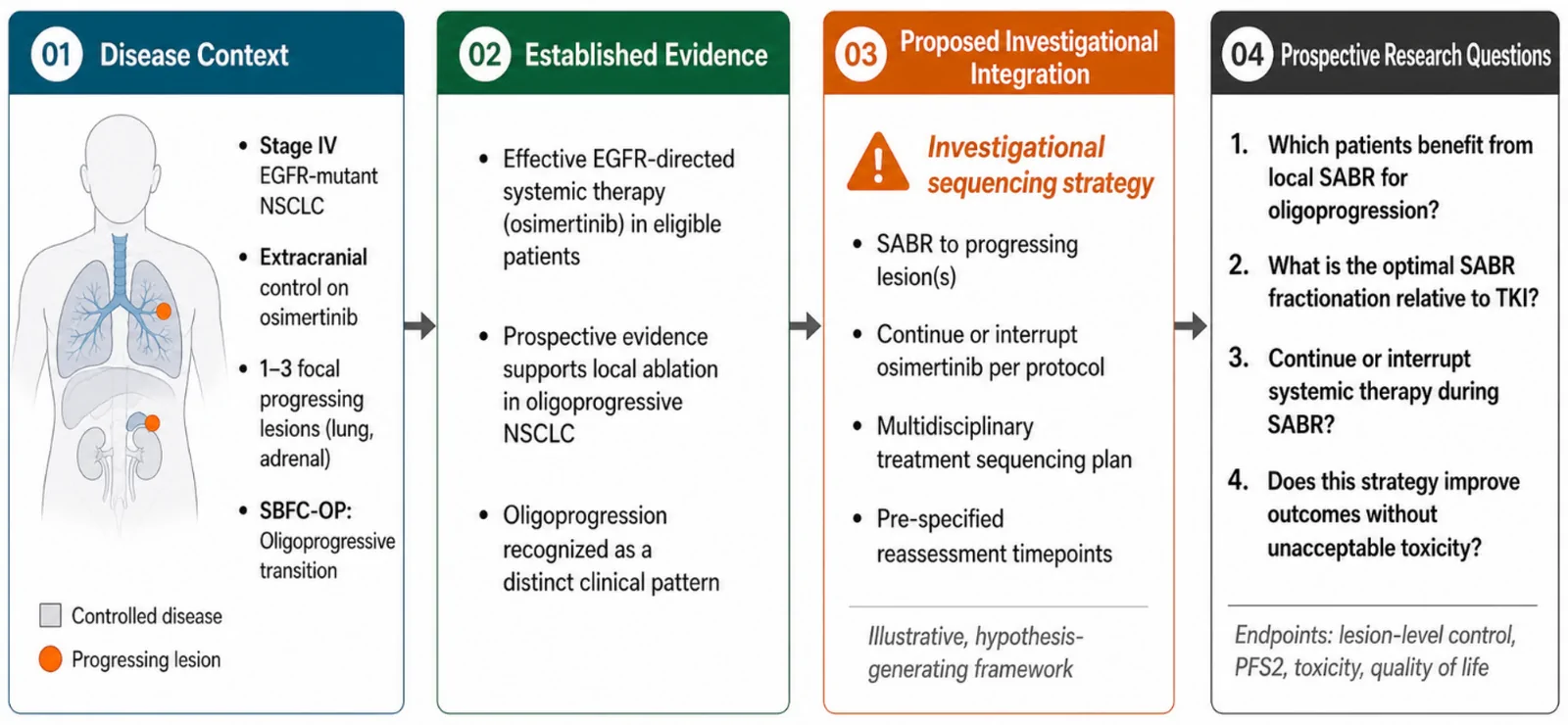
Scenario A: EGFR-Mutant Non-Small Cell Lung Cancer with Oligoprogression (SBFC-OP). Illustrative research framework for EGFR-mutant non-small cell lung cancer with oligoprogression. In patients with extracranial disease control on osimertinib and 1–3 progressing lesions, local SABR may be evaluated while systemic therapy is continued or interrupted according to protocol. Prospective studies should define patient selection, SABR timing and dose fractionation, systemic-therapy sequencing, clinically meaningful outcomes, and toxicity. This figure presents a proposed research framework rather than a clinical practice mandate. Abbreviations: EBRT, external-beam radiotherapy; EGFR, epidermal growth factor receptor; NSCLC, non-small cell lung cancer; SABR, stereotactic ablative radiotherapy; SBFC-OP, oligoprogressive transition.

**Figure 4:**
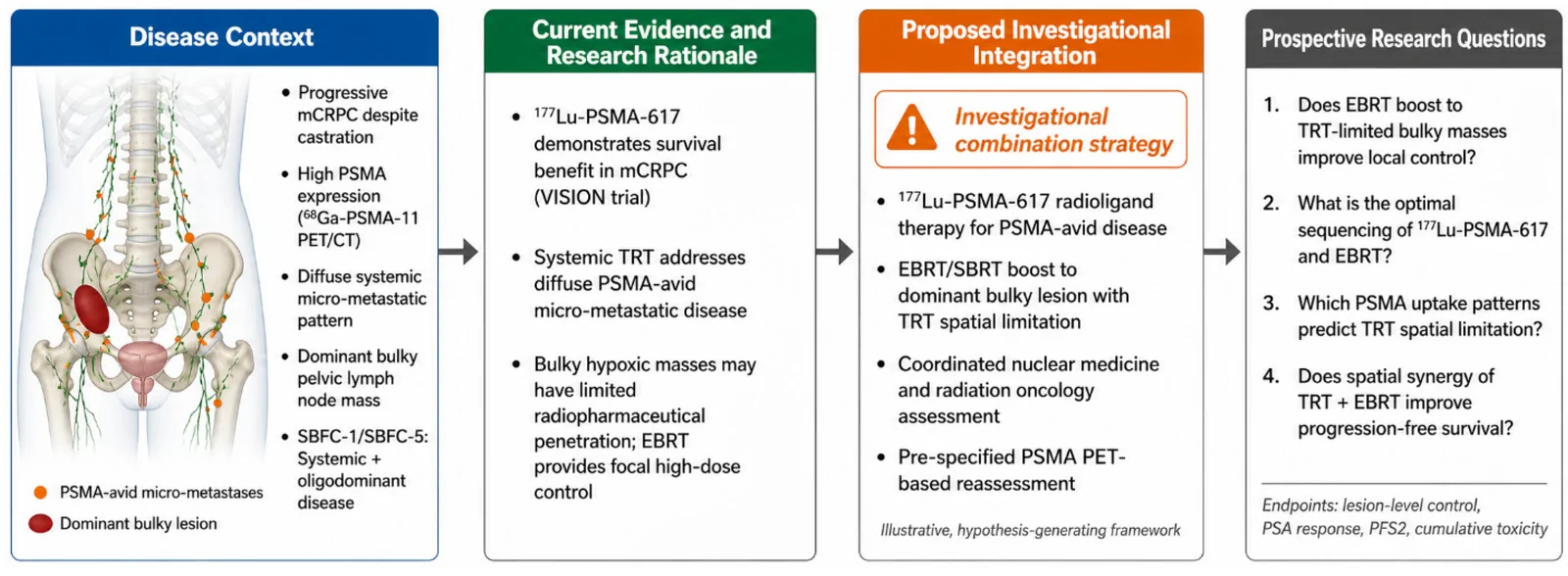
Scenario B: Metastatic Castration-Resistant Prostate Cancer—Investigational TRT–EBRT Integration (SBFC-5/SBFC-TINV). Illustrative research framework for metastatic castration-resistant prostate cancer (mCRPC) with diffuse PSMA-avid disease and a dominant bulky pelvic lesion. ^177^Lu-PSMA-617 has established evidence for appropriately selected patients with PSMA-expressing mCRPC. The addition and sequencing of focal external-beam radiotherapy for a bulky or incompletely responding lesion remain investigational. Prospective studies should define patient selection, treatment timing, cumulative TRT–EBRT dosimetry, organ-at-risk constraints, hematologic safety, and comparative clinical benefit.

**Figure 5:**
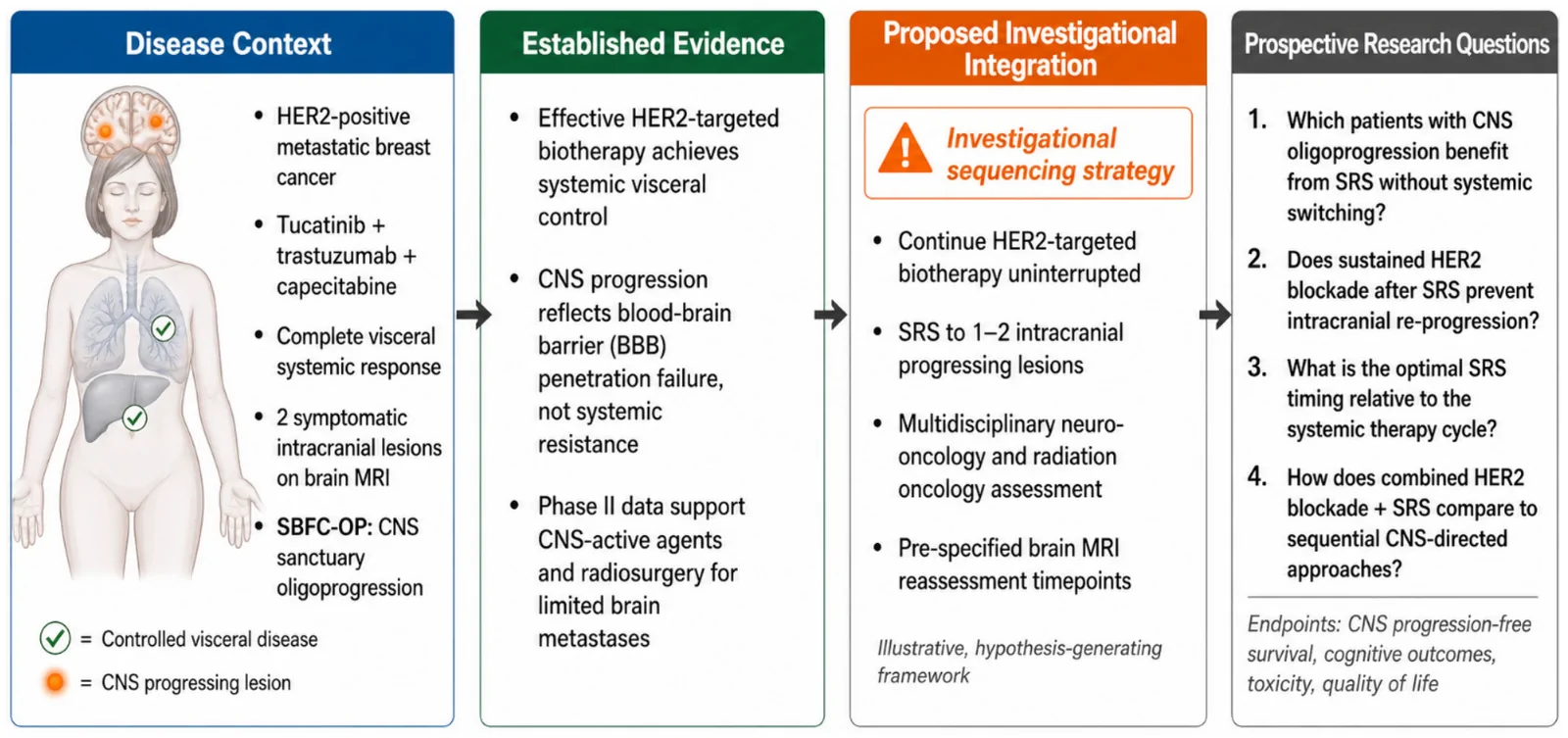
Scenario C: HER2-Positive Breast Cancer with CNS-Dominant Progression (SBFC-CNS). Illustrative research framework for HER2-positive metastatic breast cancer with CNS-dominant progression despite extracranial disease control. In the illustrated scenario, two small symptomatic brain metastases develop during treatment with tucatinib, trastuzumab, and capecitabine. Focal stereotactic radiosurgery may be evaluated while the HER2-directed systemic regimen is continued or briefly interrupted according to protocol, agent-specific safety guidance, and multidisciplinary assessment. Prospective studies should define patient selection, treatment timing, intracranial control, neurologic toxicity, systemic disease control, and quality of life. This figure presents a proposed research framework rather than a clinical practice mandate.

**Figure 6:**
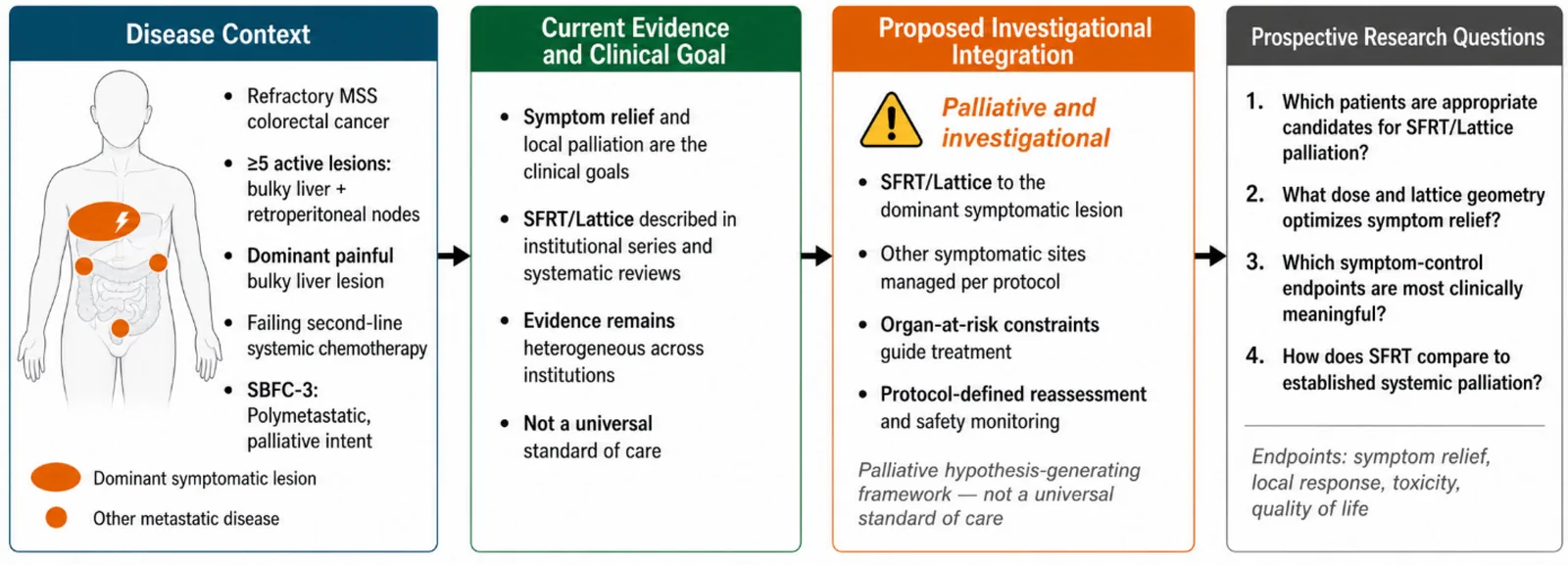
Scenario D: Colorectal Cancer with Polymetastatic Bulky Recurrence (SBFC-3/SBFC-SyBP). Illustrative research framework for refractory microsatellite-stable colorectal cancer with polymetastatic progression and a dominant symptomatic bulky liver lesion. Spatially fractionated radiotherapy (SFRT/Lattice) may be evaluated for palliation of the dominant lesion, while other symptomatic sites are managed according to clinical need, organ-at-risk constraints, and protocol-defined intent. Evidence remains heterogeneous and does not establish a universal standard of care. Prospective studies should define patient selection, dose and lattice geometry, symptom-control endpoints, toxicity, and comparative value relative to established palliative approaches. This figure presents a palliative, hypothesis-generating research framework rather than a clinical practice mandate.

To demonstrate the practical utility of the State-Based Framework Categorization (SBFC), we outline four clinical scenarios across major disease sites. These scenarios translate established concepts in oligometastatic and oligoprogressive disease, response-adaptive treatment, and cross-modality radiotherapy into illustrative multidisciplinary decision loops [1,14,15,22]. All clinical scenarios, decision thresholds, and treatment combinations described here are hypothesis-generating research frameworks for prospective trial design rather than mandatory clinical practice guidelines [5]. Figure 3, Figure 4, Figure 5 and Figure 6 show how treatment intent, modality selection, and sequencing may adapt to differences in tumor biology, disease burden, spatial distribution, and clinical trajectory.

### 4.1. Scenario A: EGFR-Mutant Non-Small Cell Lung Cancer with Oligoprogression (SBFC-OP)

Clinical Setting & Baseline: A patient with stage IV EGFR-mutant NSCLC on first-line osimertinib achieves systemic disease control but develops 1 to 3 PET-avid, focal progressing lesions in the lung and adrenal gland (SBFC-OP) [33,35].

Integrated Intervention: Local stereotactic ablative radiotherapy (SABR) may be evaluated for the progressing lesions while osimertinib is continued or interrupted according to protocol, agent-specific safety guidance, and multidisciplinary assessment [34]. Prospective studies should determine appropriate patient selection, treatment timing, and dose fractionation.

Biological & Clinical Rationale: Oligoprogression may reflect limited focal progression while disease elsewhere remains controlled. Local SABR may be evaluated to control progressing lesions and potentially prolong benefit from an otherwise effective systemic regimen; whether this strategy improves clinically meaningful outcomes without unacceptable toxicity requires prospective study [33].

Evidence Status and Research Question: Prospective phase II and randomized evidence supports local consolidative therapy in selected patients with oligoprogressive or limited metastatic NSCLC receiving effective systemic therapy [32,33,34,35,44,45]. Further prospective studies should define which patients benefit, the optimal timing and dose of SABR, whether systemic therapy should be continued or interrupted, and whether this strategy improves clinically meaningful outcomes without unacceptable toxicity.

### 4.2. Scenario B: Metastatic Castration-Resistant Prostate Cancer (mCRPC)

#### Investigational TRT–EBRT Integration (SBFC-TINV)

Clinical Setting & Baseline: A patient with progressive mCRPC exhibits diffuse, high PSMA expression on ^68^Ga-PSMA-11 PET/CT together with a dominant bulky pelvic lymph node mass threatening the adjacent ureter [17,54].

Integrated Intervention: The patient receives ^177^Lu-PSMA-617 for systemic PSMA-avid disease [17]. Focal external-beam radiotherapy to the dominant bulky pelvic lesion may be evaluated as an investigational addition; the timing, sequencing, cumulative dosimetry, and safety of the combined approach remain to be defined prospectively [48,49,50].

Biological & Clinical Rationale: ^177^Lu-PSMA-617 provides systemic treatment for PSMA-expressing disease, whereas focal EBRT may be investigated for a bulky or incompletely responding lesion. The potential value, timing, cumulative dosimetry, and safety of this integration remain uncertain and require prospective evaluation [50].

Evidence Status and Research Question: ^177^Lu-PSMA-617 has established evidence for appropriately selected patients with metastatic castration-resistant prostate cancer [17]. The addition and sequencing of focal EBRT for bulky or incompletely responding lesions remains investigational, supported by radiobiological rationale, review-level synthesis, and early feasibility data [15,48,49,50,51,52,53]. Prospective studies should define patient selection, treatment sequence, cumulative dosimetry, organ-at-risk constraints, hematologic safety, and comparative clinical benefit.

### 4.3. Scenario C: HER2-Positive Breast Cancer with CNS-Dominant Progression (SBFC-CNS)

#### Proposed CNS-Dominant Progression Research Framework

Clinical Setting & Baseline: A patient with HER2-positive metastatic breast cancer receiving tucatinib, trastuzumab, and capecitabine shows complete systemic response in visceral organs but develops two small, symptomatic parenchymal brain metastases on MRI [55].

Biological & Clinical Rationale: CNS-dominant progression despite extracranial disease control does not necessarily indicate generalized resistance to systemic HER2-directed therapy. Focal SRS may control limited gross intracranial disease while allowing continued use of an otherwise effective systemic regimen, subject to protocol-defined safety guidance and multidisciplinary assessment [56].

Integrated Intervention: Systemic biotherapy is continued or briefly interrupted according to the study protocol, agent-specific safety guidance, and multidisciplinary assessment, while stereotactic radiosurgery (SRS, 18–24 Gy in a single fraction or 24 Gy in 3 fractions) is delivered to the two intracranial lesions [57].

Evidence Status and Research Question: HER2CLIMB supports CNS-active tucatinib, trastuzumab, and capecitabine [55], while retrospective clinical experience and an ongoing prospective protocol address integration with stereotactic radiosurgery [56,57]. Prospective studies should define patient selection, SRS timing, whether systemic therapy should be continued or briefly interrupted, intracranial control, neurologic toxicity, systemic disease control, and quality of life.

### 4.4. Scenario D: Colorectal Cancer with Polymetastatic Bulky Recurrence (SBFC-3/SBFC-SyBP)

Clinical Setting & Baseline: A patient with refractory microsatellite-stable (MSS) colorectal cancer presents with painful, bulky liver and retroperitoneal nodal progression (≥5 active lesions) failing second-line systemic chemotherapy [46,47].

Integrated Intervention: Spatially fractionated radiotherapy (SFRT/Lattice) may be evaluated for palliation of the dominant symptomatic liver lesion. Other symptomatic sites are managed according to clinical need, organ-at-risk constraints, and protocol-defined intent [26,43,46,47].

Biological & Clinical Rationale: The intent is palliative symptom relief and local debulking (SBFC-3). SFRT/Lattice creates heterogeneous peak-and-valley dose distributions intended to treat bulky tumors while limiting dose to surrounding normal tissues; proposed radiobiological mechanisms and clinical outcomes remain areas of active study [26,43,46,47].

Evidence Status and Research Question: Institutional series and systematic reviews describe SFRT/Lattice as a palliative option for selected bulky, refractory tumors, including gastrointestinal malignancies, but the evidence is heterogeneous and does not establish a universal standard of care [26,43,46,47]. Prospective studies should define suitable patient selection, dose and geometric parameters, symptom-control endpoints, toxicity, integration with systemic therapy, and comparative value relative to established palliative approaches.

## 5. Toxicity Monitoring, Radiobiological Considerations, and Future Directions

Translating the IRBT framework from disease-specific applications into safe, reproducible clinical investigation requires an equally integrated approach to toxicity surveillance, cumulative radiobiological dosimetry, and the evidentiary, educational, and policy priorities that will shape its future development.

### 5.1. Provisional Toxicity Monitoring Protocols

Combining external-beam radiotherapy, targeted radiopharmaceuticals, and systemic biotherapies creates overlapping toxicity profiles that require proactive monitoring beyond the acute mucosal and hematologic toxicities emphasized in historical chemoradiation.

Integrated radiobiotherapy may require serial assessment of hematologic status, organ function, and organ-at-risk effects because EBRT, therapeutic radiopharmaceuticals, and systemic agents can produce overlapping toxicities [15,28,29,51,58,59]. Potential protocol assessments include complete blood counts with absolute lymphocyte count, metabolic panels, agent-specific organ-function studies, and quantitative imaging or dosimetry. The frequency of testing, duration of follow-up, and treatment-hold thresholds must be defined for each agent-radiation combination using applicable trial protocols, product information, practice parameters, and prospective safety data. Any numerical schedules or thresholds used in future IRBT studies should therefore be treated as protocol-specific, hypothesis-generating parameters rather than universal clinical requirements [15,28,29,59].

### 5.2. Cumulative Radiobiological Dosimetry (TRT + EBRT)

Evaluating cumulative normal-tissue and tumor doses when combining continuous, low-dose-rate targeted radionuclide therapy with acute, high-dose-fraction EBRT presents substantial radiobiological complexity [15,51,52,53,58,60]. Simple physical dose summation is inadequate because the modalities differ in dose rate, spatial distribution, repair kinetics, and temporal protraction [51,52,53,58,60]. Biological comparisons may use BED or EQD2 approaches, but radionuclide applications require models that account for dose rate, decay, and tissue repair rather than direct transfer of fractionated-EBRT assumptions [52,53,58,60,61].

Supplementary File S3 provides the mathematical derivations, parameter definitions, and sensitivity analyses needed to support transparent design and reporting of combined TRT–EBRT dosimetry studies.

For conventionally fractionated EBRT, the linear-quadratic model expresses biologically effective dose as BED = D [1 + d/(α/β)], where D is the total absorbed dose, d is the dose per fraction, and α/β is the tissue-specific radiosensitivity parameter [61]. Application to targeted radionuclide therapy requires additional modeling of dose rate, radionuclide decay, spatial nonuniformity, and repair kinetics [52,53,58,60].

Cumulative organ-at-risk modeling must explicitly report parameter assumptions and uncertainty, because changes in α/β, fraction size, dose rate, and repair kinetics can materially alter biological dose estimates [52,61,62].

Fixed α/β values should not be treated as absolute constants. Prospective trials combining TRT and EBRT should incorporate quantitative patient-specific imaging and dosimetry, with voxel-level reconstruction used when technically feasible and appropriate to the study question [15,52,53,59,60,63].

### 5.3. Policy, Educational, and Research Next Steps

To advance Integrated Radiobiotherapy from a conceptual framework toward prospective validation and potential future clinical implementation, four strategic priorities should be pursued across health policy, research infrastructure, and cross-specialty clinical domains [59].

Prospective Clinical Trial Design: Multi-institutional trials should evaluate state-based adaptation rules—such as local consolidation for oligoprogression versus systemic-therapy switching—using disease-control, quality-of-life, safety, and survival endpoints [32,36,44,45].

Interdisciplinary Training Programs: Radiation oncology and related training programs should expand education in molecular theranostics, systemic-therapy interactions, radiation safety, quantitative imaging, and radiobiological modeling [63,64,65].

Standardized Reporting & QA: Radiation oncology and nuclear medicine societies should develop harmonized standards for radiopharmaceutical-therapy performance, cumulative EBRT-TRT dosimetry, spatial response reporting, and trial eligibility [15,59,63,64,66].

Regulatory & Payment Innovation: Payers and regulatory agencies should evaluate care-delivery and reimbursement models that can accommodate coordinated multimodality treatment while preserving evidence, safety, and value requirements [66,67,68].

## 6. Broader Considerations & Policy Dimensions

Moving Integrated Radiobiotherapy (IRBT) from an academic proposal into clinical investigation requires attention to regulation, quality assurance, access, and reimbursement. Oncology care-delivery and payment models can create administrative barriers to coordinated multimodality care, supporting evaluation of alternative and bundled approaches where evidence and policy permit [67,68]. Professional practice parameters and broader theranostics policy initiatives provide a foundation for safe program development [59,66]. International trial design must also account for unequal access to radiotherapy, theranostic infrastructure, trained personnel, and approved agents [66,69]. These policy proposals remain recommendations for evaluation rather than established reimbursement or regulatory standards.

## 7. Historical Context & the Two Crossroads

The development of Integrated Radiobiotherapy can be understood through two earlier crossroads in radiation medicine. The first was technological: in 1995, the implementation of three-dimensional conformal radiation therapy demonstrated that advances in treatment precision required more than new equipment; they also required rigorous quality assurance, standardized planning, multidisciplinary training, and reproducible clinical workflows [70]. The second was conceptual: in the same year, Hellman and Weichselbaum proposed the oligometastatic state as an intermediate biological condition between localized and widely metastatic disease, creating a rationale for investigating metastasis-directed treatment in carefully selected patients [71]. Together, these contributions anticipated the central challenge addressed by IRBT: how to translate increasingly precise radiation delivery and increasingly nuanced disease-state classification into coordinated, prospectively testable treatment strategies. Contemporary molecular imaging, targeted radionuclide therapy, adaptive external-beam radiotherapy, and systemic biotherapies now create a new implementation crossroads, but the historical lesson remains applicable. Technical capability alone does not establish clinical value, and a compelling biological concept does not substitute for validation. Rigorous quality assurance, standardized terminology and endpoints, multidisciplinary governance, and prospective trials are therefore necessary to determine whether the proposed state-based IRBT framework improves outcomes, safety, feasibility, or value.

## 8. Conclusions

Integrated Radiobiotherapy (IRBT) proposes a dynamic, state-based framework for examining the relationship between static anatomical staging and response-adaptive oncology. By organizing prospective investigation around explicit biological and anatomical variables—target expression, spatial heterogeneity, systemic burden, and organ-at-risk safety constraints—the framework may support more coordinated evaluation of multidisciplinary treatment sequences. IRBT is intended as an educational model, operational roadmap, and hypothesis-generating research agenda for clinical trial design rather than a clinical practice mandate. Prospective studies and improved cross-modality dosimetry will be required to determine whether this approach improves clinical outcomes, safety, feasibility, or value.

## Figures and Tables

**Table 1:** Terminology, definitions, and conceptual distinctions.

Terminology	Operational Definition	Clinical Problem Addressed	Distinction from Existing Modalities/Scope
Radiobiotherapy	Deliberate biologically synchronized integration of radiation with modern systemic biotherapies (e.g., targeted therapies, ICIs)	Addresses historical default pairing with non-specific cytotoxic chemoradiation	Focuses on biological and immunological synergy rather than broad radiosensitization or toxic overlapping cell kill
Integrated Radiobiotherapy (IRBT)	Proposed state-guided research framework using shared selection criteria, dynamic state-based transitions, and prespecified adaptation rules	Provides a structure for studying coordination across specialty handoffs and limitations of static stage-bound decision-making in multisite disease	Coordinates interdisciplinary modalities such as SABR, SFRT, TRT, and systemic therapy within a proposed state-guided research framework
Radiobiomedicine	Overarching academic and clinical discipline encompassing biology-guided, tissue-sparing physical radiation across both oncologic and non-malignant conditions	Advances cancer care through integrated, biology-based decision-making across the care continuum	Serves as master discipline under which IRBT (oncologic) and FRM (non-oncologic) reside

Note: Radiobiomedicine was first formally defined in a 2020 publication [4].

## Data Availability

No new data were created or analysed in this study. Data sharing is not applicable to this article.

## Supplementary Materials

The following supporting information can be downloaded at: https://www.mdpi.com/article/10.3390/curroncol33100637/s1, File S1: Expanded Legend, Glossary, and Conceptual Framework Reference; File S2: State-Based Framework Categorization (SBFC) Clinical Worksheet; File S3: Cumulative TRT–EBRT Dosimetry Calculation Framework and Sensitivity Protocol; File S4: International Prospective Registry Protocol Outline (Global Integrated Radiobiotherapy and Radiobiomedicine Registry [G-IRBT Registry]: A Multi-Center Prospective Observational Study of State-Based Response-Adaptive Radiation Sequencing). Main-manuscript references are numbered [1,2,3,4,5,6,7,8,9,10,11,12,13,14,15,16,17,18,19,20,21,22,23,24,25,26,27,28,29,30,31,32,33,34,35,36,37,38,39,40,41,42,43,44,45,46,47,48,49,50,51,52,53,54,55,56,57,58,59,60,61,62,63,64,65,66,67,68,69,70,71], and supplementary-only references are labeled [72,73,74,75,76,77,78,79,80,81,82,83,84,85]. All references are included together in the global reference list; the relevant S-prefixed references are repeated within the applicable supplementary files for reader convenience.

## Abbreviations

Terminology Note: Targeted radionuclide therapy (TRT) is used throughout this manuscript as the umbrella term for systemically administered, molecularly targeted radiopharmaceutical therapy. The more specific terms radioligand therapy (RLT) and peptide receptor radionuclide therapy (PRRT) are retained only when required by the cited literature or by the therapeutic platform under discussion.

Abbreviations	Expansion
AJCC	American Joint Committee on Cancer
ALC	Absolute Lymphocyte Count
EBRT	External-Beam Radiotherapy
FDG	Fluorodeoxyglucose (^18^F-FDG, used in PET imaging)
ICI	Immune Checkpoint Inhibitor(s)
IMRT	Intensity-Modulated Radiation Therapy
IRBT	Integrated Radiobiotherapy
Lattice RT	Lattice Radiotherapy (a form of spatially fractionated radiotherapy)
MDT	Multidisciplinary Team/Decision
MDT-RT	Metastasis-Directed Therapy using Radiotherapy
OAR	Organ(s) at Risk
PET	Positron Emission Tomography
PRRT	Peptide Receptor Radionuclide Therapy (variant term; see TRT)
PSMA	Prostate-Specific Membrane Antigen
QA	Quality Assurance
RFI	Recurrence-Free Interval
RLT	Radioligand Therapy (variant term; see TRT)
SABR	Stereotactic Ablative Radiotherapy (used when emphasizing ablative intent)
SBRT	Stereotactic Body Radiation Therapy (the precise, image-guided extracranial delivery technique)
SFRT	Spatially Fractionated Radiotherapy
SSR PET	Somatostatin Receptor PET (e.g., ^68^Ga-DOTATATE)
TNM	Tumour–Node–Metastasis (staging classification)
TRT	Targeted Radionuclide Therapy (umbrella term; includes agent-specific RLT and PRRT)
