# Supplementary material for: Integrated Radiobiotherapy: A State-Based Framework for Response-Adaptive Combination Oncology
Source: Curr Oncol. 2026 Oct 20;33(10):637. doi: 10.3390/curroncol33100637 (PMC13703004; doi:10.3390/curroncol33100637)
Supplement: Supplementary file 1 [file curroncol-33-00637-s001.zip › curroncol-4486443-supplementary.pdf]

Supplementary File S1: Expanded Legend, Glossary, and Conceptual Framework Reference

Manuscript: Integrated Radiobiotherapy: A State-Based Framework for Response-Adaptive Combination Oncology

Journal: *Current Oncology*

Part A: Expanded Legend for Figure 1

Figure 1. Integrated Radiobiotherapy (IRBT) as a State-Based Decision Loop — from stage-based handoffs to state-based, response-adaptive integration of molecular imaging, systemic therapy, theranostics, and external-beam radiotherapy.

Left Panel — Stage-Based Care

The left panel depicts the conventional oncology care pathway, in which a one-time anatomical staging assessment (TNM/AJCC) assigns patients to a fixed disease category that determines the sequence of specialty-specific treatment blocks — medical oncology, radiation oncology, and surgery — operating as siloed decision units with unidirectional handoffs and no structured feedback loop. Reassessment is ad hoc and reactive rather than pre-specified. Patients presenting with identical stage IV diagnoses may exhibit vastly different clinical trajectories depending on driver mutation status, PD-L1 expression, circulating tumour DNA dynamics, and microenvironmental heterogeneity — a granularity that anatomical staging alone cannot capture.

Central Transition

The central arrow represents the conceptual shift proposed by IRBT: from static stage-bound decision-making toward a research framework using shared selection criteria, dynamic state-based reassessment, and prespecified adaptation rules. This transition is intended for prospective

evaluation and does not replace established staging systems, multidisciplinary judgment, or standard clinical pathways.

Right Panel — State-Based Integrated Radiobiotherapy (IRBT)

The right panel depicts a proposed five-step iterative research loop. Each step is described below using its functional label.

Step 1 — Clinical State Assessment

The primary input to the loop and the foundational construct of IRBT. Rather than a one-time staging event, state assessment is a continuous, pre-specified re-evaluation of four interacting variables that together define an actionable biological and anatomical operational window:

(a) Target Expression and Molecular Imaging Phenotype. The density, distribution, and functional activity of therapeutic targets as measured through molecular imaging—including PSMA PET, ^{18}F-FDG PET, and somatostatin-receptor PET—or tissue-based biomarkers. This variable informs eligibility for targeted radionuclide therapy (TRT) and the biological rationale for EBRT field design.

(b) Spatial Heterogeneity & Lesion Geometry. The physical distribution of disease — focal versus bulky, hypoxic core versus vascularized periphery — which governs whether uniform ablative SBRT/SABR or spatially fractionated techniques (SFRT/Lattice RT) are more appropriate, and whether selective field reduction or dose painting is feasible.

(c) Systemic Tumour Burden & Tempo. The absolute lesion count (oligometastatic versus polymetastatic) and the rate of disease progression over time (recurrence-free interval, RFI), which together determine whether therapeutic intent is consolidative or palliative, and whether metastasis-directed therapy (MDT-RT) is appropriate to extend benefit from an active systemic regimen.

(d) Organ-at-Risk & Hematologic Constraints. Normal-tissue tolerance limits, prior radiation dose overlap, and baseline immune status—including absolute lymphocyte count (ALC)—define safety considerations that must be operationalized through disease-, agent-, and protocol-specific eligibility criteria, dose constraints, monitoring schedules, and treatment-modification rules.

Step 2 — Multidisciplinary Decision

Synthesis of the current clinical state by a multidisciplinary team at prespecified reassessment timepoints. Within prospective studies, this step may integrate oncologic, radiobiological, and patient-specific factors into a protocol-defined treatment strategy and sequencing plan.

The *Oligometastatic/Oligoprogressive Spectrum* callout identifies a clinically relevant setting for prospective evaluation of EBRT, TRT, and systemic therapy. Studies may examine whether metastasis-directed radiotherapy can control oligoprogressive lesions while an otherwise effective systemic regimen is continued or interrupted according to protocol; whether lesion-level response after TRT can identify residual or clinically dominant sites for focal EBRT; and whether multidisciplinary sequencing plans with prespecified reassessment timepoints improve outcomes, safety, feasibility, or value.

Step 3 — Therapy Selection

Selection of one or more modalities based on the current clinical state and multidisciplinary consensus, from three coordinated therapeutic options:

(a) Systemic Therapy. Chemotherapy, targeted agents, or immune checkpoint inhibitors may be selected according to molecular profile, prior treatment exposure, disease state, and applicable standards. Prospective IRBT studies should define how systemic therapy is continued, interrupted, or sequenced with EBRT or TRT and should evaluate potential biological interaction, cumulative toxicity, and clinically meaningful outcomes.

(b) TRT—Targeted Radionuclide Therapy. Systemically administered radiopharmaceuticals deliver radiation to molecularly defined targets demonstrated by imaging or other validated biomarkers. TRT may be selected when target expression, disease distribution, treatment history, organ function, and protocol-specific safety criteria support its use. Agent-specific terms such as radioligand therapy (RLT) and peptide receptor radionuclide therapy (PRRT) are retained only when required by the cited literature or therapeutic platform.

(c) EBRT — External-Beam Radiotherapy. Delivered as stereotactic body radiation therapy (SBRT), with stereotactic ablative radiotherapy (SABR) used when emphasizing ablative intent; spatially fractionated radiotherapy (SFRT/Lattice RT); or conventionally fractionated intensity-modulated radiation therapy (IMRT). Modality and intent are selected according to lesion geometry, spatial heterogeneity, and dose constraints established during Clinical State Assessment. EBRT may serve a consolidative, ablative, or palliative role depending on the current state and is coordinated—not siloed—within the broader sequencing plan.

Step 4 — Delivery & Quality Assurance

Execution of the selected therapy with concurrent quality assurance processes, including dosimetric verification, image-guided delivery, and real-time enforcement of safety boundaries for normal tissue and hematologic tolerance. This step operationalizes the safety constraints established in Clinical State Assessment and ensures that delivery fidelity matches the biological intent of the selected approach.

Step 5 — Reassessment & Adaptation

Structured re-evaluation at a pre-specified timepoint, generating the updated clinical state that re-enters Step 1 and closes the loop. Four adaptation decisions are available at this step:

- **Escalate** — increase dose or volume to focal sites of resistance identified on reassessment imaging.
- **Consolidate** — apply protocol-defined local or systemic consolidation when reassessment confirms response or identifies residual disease for which consolidation is justified.
- **Switch** — transition to an alternative modality (e.g., from EBRT to TRT when systemic burden increases or target expression is confirmed on follow-up PET; or from systemic therapy to MDT-RT at oligoprogression).
- **De-escalate** — reduce volume, fraction size, or treatment intensity to prevent cumulative lymphopenia, excess organ-at-risk exposure, or immune compromise.

The feedback arrow from *Reassessment & Adaptation* back to *Clinical State Assessment*, labeled *Pre-specified reassessment node*, represents the framework's proposed iterative feature. Prospective studies should determine whether this structure improves decision consistency, safety, feasibility, clinical outcomes, or value compared with standard multidisciplinary care.

Part B: Abbreviations & Terminology

Abbreviation Table

Abbreviation	Expansion
AJCC	American Joint Committee on Cancer
ALC	Absolute Lymphocyte Count
EBRT	External-Beam Radiotherapy
FDG	Fluorodeoxyglucose (^{18}F-FDG, used in PET imaging)
ICI	Immune Checkpoint Inhibitor(s)
IMRT	Intensity-Modulated Radiation Therapy
IRBT	Integrated Radiobiotherapy
Lattice RT	Lattice Radiotherapy (a form of spatially fractionated radiotherapy)
MDT	Multidisciplinary Team / Decision
MDT-RT	Metastasis-Directed Therapy using Radiotherapy
OAR	Organ(s) at Risk
PET	Positron Emission Tomography
PRRT	Peptide Receptor Radionuclide Therapy (variant term; see TRT)
PSMA	Prostate-Specific Membrane Antigen

QA	Quality Assurance
RFI	Recurrence-Free Interval
RLT	Radioligand Therapy (variant term; see TRT)
SABR	Stereotactic Ablative Radiotherapy (used when emphasizing ablative treatment intent)
SBFC	State-Based Framework Categorization
SBFC-OP	Oligoprogressive Transition — protocol-defined limited focal progression while disease elsewhere remains controlled (SBFC-1)
SBFC-TPCI	Oligometastatic Presentation — protocol-defined low-volume metastatic disease considered for comprehensive local treatment (SBFC-2)
SBFC-SyBP	Symptomatic Bulky Progression — palliative research setting (SBFC-3)
SBFC-AsyP	Asymptomatic High-Burden Progression — systemic-therapy-priority setting (SBFC-4)
SBFC-TINV	Investigational Cross-Modality Transition — proposed TRT–EBRT or biomarker-guided combinations (SBFC-5)
SBFC-CNS	CNS-Dominant Progression — intracranial progression despite extracranial disease control
SBRT	Stereotactic Body Radiation Therapy (the precise, image-guided extracranial delivery technique)
SFRT	Spatially Fractionated Radiotherapy
SSR PET	Somatostatin Receptor PET (e.g., ^{68}Ga-DOTATATE)
TNM	Tumour–Node–Metastasis (staging classification)
TRT	Targeted Radionuclide Therapy (umbrella term for systemically administered molecularly targeted radiopharmaceutical therapy; includes agent-specific RLT and PRRT)

Terminology Note

TRT terminology: Targeted radionuclide therapy (TRT) is used throughout the manuscript and supplementary materials as the umbrella designation for systemically administered, molecularly targeted radiopharmaceutical therapy. Agent-specific terms such as radioligand therapy (RLT) and peptide receptor radionuclide therapy (PRRT) are retained only when required by the cited literature or therapeutic platform.

Part C: Conceptual Comparison Matrix — Radiobiomedicine and Its Constituent Frameworks versus Existing Modalities

This matrix extends Table 1 of the main manuscript by comparing Radiobiomedicine, Integrated Radiobiotherapy (IRBT), Functional Radiation Medicine (FRM), conventional adaptive radiotherapy, and the traditional multidisciplinary tumor-board model across eight operational dimensions.

Table S1.1: Conceptual Comparison Matrix — Radiobiomedicine Framework vs. Existing Modalities

Dimension	Radiobiomedicine (Umbrella Discipline)	Integrated Radiobiotherapy (IRBT)	Functional Radiation Medicine (FRM)	Adaptive RT (Conventional)	Traditional Multidisciplinary Tumor Board (MDT)
**Conceptual Domain**	Master discipline encompassing biology-guided, tissue-sparing physical radiation across oncologic and non-malignant conditions. Parallel to the historical expansion of interventional radiology. [4]	Proposed research framework using shared selection criteria, dynamic state-based reassessment, and prespecified adaptation rules for prospective evaluation of oncologic multimodality integration. [5]	Non-oncologic, tissue-sparing applications of radiation for benign and inflammatory conditions using targeted cytokine, macrophage, and neuromodulatory biology. [6,9,11]	Technical adaptation of radiation treatment delivery (dose, volume, plan) during a course, driven by anatomical or geometrical changes. [S1]	Sequential specialty consultation model using one-time anatomical staging (TNM/AJCC) to assign patients to fixed treatment blocks. [19]
**Clinical Scope**	Dual: oncologic AND non-malignant. Serves as the overarching academic and clinical	Oncologic only. Multi-site, multi-modality integration of EBRT, TRT, and systemic therapy in	Non-oncologic only. Anti-inflammatory, neuromodulatory, and functional tissue	Radiation delivery only. Adapts the physical plan within a single-modality course; does	All cancer types. Sequential handoffs across radiation oncology, medical

	umbrella under which IRBT (oncologic arm) and FRM (non-oncologic arm) reside.	cancer with active or metastatic disease.	restoration in benign conditions.	not govern systemic therapy sequencing or theranostic integration.	oncology, and surgery as operationally separate disciplines.
**Target Population / Disease Setting**	Cancer patients across disease states AND patients with non-malignant, debilitating conditions (e.g., refractory osteoarthritis, cardiac arrhythmia, treatment-refractory psychiatric disorders).	Oligometastatic, oligoprogressive, bulky symptomatic, high-burden, and investigational cancer states with actionable molecular-imaging targets, active systemic regimens, or both. Six proposed SBFC categories (SBFC-0 through SBFC-5) structure prospective evaluation.	Patients with non-malignant conditions requiring tissue-targeted radiation: degenerative joint disease, refractory atrial fibrillation, refractory obsessive-compulsive disorder or depression, spinal pain.	Any patient undergoing a radiotherapy course with measurable anatomical change (e.g., head-and-neck cancer with tumor shrinkage, GU cancer with daily bladder/rectal filling variation).	All newly diagnosed or recurrent cancer patients requiring coordinated specialty input.
**Core Decision Logic**	Biology-guided, mechanism-based. Sub-millimeter spatial precision is paired with targeted	State-Based Framework Categorization (SBFC). Four interacting clinical state variables — target expression,	Mechanism-based, functional outcome–driven. Decisions target cytokine, macrophage,	Anatomy-driven, geometry-based. Treatment plan adaptation is triggered by measurable	Stage-based (TNM/AJCC). One-time anatomical classification drives the long-term treatment sequence.

	biological effects: cytokine modulation (LDRT), immune priming (SBRT), or theranostic targeting (TRT). Decisions are state-informed at both the oncologic and non-oncologic levels.	spatial heterogeneity, systemic tumour burden/tempo, and OAR/hematologic constraints — drive a five-step iterative decision loop with pre-specified adaptation rules.	or neural pathway modulation rather than tumor kill. Not governed by SBFC.	volumetric or positional change detected on imaging (CBCT, MRI). No systemic or molecular state integration.	Specialty decisions are made independently; reassessment is reactive, not pre-specified.
**Key Technologies / Modalities**	SBRT/SABR, SFRT/Lattice RT, LDRT, stereotactic radiosurgery (SRS), TRT (PSMA-targeted, PRRT, RLT), molecular PET imaging (PSMA PET, FDG PET, SSR PET), MR-guided delivery.	SBRT/SABR (consolidative, ablative); SFRT/Lattice RT (bulky disease); TRT (PSMA Lu-177, ^{68}Ga-DOTATATE, Ac-225 agents); ICI and targeted systemic therapy; PSMA/SSR/FDG PET for state reassessment.	LDRT (typically 0.5–2 Gy per fraction); cardiac radiosurgery (ENCORE protocol); radiosurgical capsulotomy (SRS-guided); spinal cord SRS for refractory pain syndromes.	CBCT-guided online adaptive RT; MR-Linac (online MRI-guided plan adaptation); offline re-planning triggered by volumetric change thresholds.	Conventional RT fractionation; standard chemotherapy regimens; surgery (resection, reconstruction); cross-sectional CT/MRI anatomical imaging.
**Reassessment Approach**	IRBT arm: structured, pre-specified reassessment nodes at defined clinical timepoints. FRM arm: functional outcome–guided re-	Structured, pre-specified reassessment nodes embedded in the five-step loop. Reassessment at Step 5 generates the updated	Functional response endpoints; reassessment driven by patient-reported outcomes, symptom scores, or objective	Imaging-based within each fraction (online ART) or at defined intervals (offline ART). Confined to	Ad hoc and reactive. Reassessment is triggered by clinical change (progression, new symptoms), not pre-specified. No

	evaluation. Both arms replace reactive reassessment with pre-planned reassessment intervals.	clinical state re-entering Step 1. Four adaptation decisions available: escalate, consolidate, switch, de-escalate.	functional measures (e.g., joint range of motion, arrhythmia recurrence).	dose/volume adaptation; does not integrate broader disease state reassessment.	structured feedback loop between treatment delivery and decision architecture.
**Treatment Intent**	State-determined. Curative (IRBT arm, early-state disease); disease-control and survival extension (IRBT arm, advanced disease); symptom control, functional restoration, and non-malignant disease modification (FRM arm).	State-informed and protocol-defined: observation for SBFC-0; evaluation of consolidative or ablative approaches in selected SBFC-1 and SBFC-2 settings; palliative approaches for SBFC-3 and SBFC-4; and investigational cross-modality combinations for SBFC-5.	Disease modification and functional restoration in non-malignant conditions. Not curative intent in the oncologic sense; goals are symptom reduction, joint preservation, arrhythmia elimination, or psychiatric symptom relief.	Maximize dose conformity and minimize normal tissue exposure within an active course. Does not independently determine or modify systemic treatment intent.	Determined by anatomical stage at diagnosis. Treatment intent (curative vs. palliative) is assigned once and updated only reactively when disease status changes clinically.
**Current Evidence Status**	Emerging academic discipline. Individual constituent approaches have modality-	Hypothesis-generating framework. Individual components—including oligometastati	Emerging. Clinical investigation spans low-dose radiotherapy for	Mature and commercially deployed. Supported by multiple prospective trials and institutional series (MR-	Standard of care. Supported by decades of modality-specific RCT evidence. MDT format recommended

	specific evidence, whereas their integration under a single Radiobiomedicine framework remains conceptual and hypothesis-generating [4,6,9,11].	c SABR, targeted radionuclide therapy, and adaptive EBRT—have clinical or technical support, but fully coordinated, response-adaptive multimodality integration has not been validated in prospective randomized trials [14–15,17–18,52–53].	inflammatory conditions and stereotactic approaches for selected functional indications; these applications require indication-specific evidence, governance, and safety evaluation [6,9,11].	Linac programs, head-and-neck ART trials). Standard workflow at high-volume academic centers. [S1]	by major oncology guidelines (NCCN, ESMO, ASTRO); patient and family participation in tumor conferences improves patient-focused cancer care. [S2,S6]

Abbreviations: AJCC, American Joint Committee on Cancer; CBCT, cone-beam CT; EBRT, external-beam radiotherapy; FDG, fluorodeoxyglucose; FRM, Functional Radiation Medicine; ICI, immune checkpoint inhibitor; IRBT, Integrated Radiobiotherapy; LDRT, low-dose radiotherapy; MDT, multidisciplinary team/decision; OAR, organ(s) at risk; PET, positron emission tomography; PRRT, peptide receptor radionuclide therapy; PSMA, prostate-specific membrane antigen; RCT, randomized controlled trial; RLT, radioligand therapy; SABR, stereotactic ablative radiotherapy; SBFC, State-Based Framework Categorization; SBRT, stereotactic body radiation therapy; SFRT, spatially fractionated radiotherapy; SRS, stereotactic radiosurgery; SSR, somatostatin receptor; TRT, targeted radionuclide therapy; TNM, Tumour–Node–Metastasis.

Part D: Evidence Milestones at the Second Crossroads

Table S1.2 summarizes selected technical, biological, and regulatory milestones that illustrate the contemporary inflection point described in Section 7 of the main manuscript. The table is intended as contextual synthesis rather than a comprehensive chronology or evidence hierarchy.

Table S1.2: Selected Milestones at the Second Crossroads—Five Domains Reshaping Radiation Medicine

Domain	Key Milestone	Year	Significance
**Radiotheranostic Approvals**	FDA approval of ^{177}Lu-DOTATATE (Lutathera) for gastroenteropancreatic neuroendocrine tumors	2018	First regulatory validation of the theranostic paired-agent model (image-then-treat with same target); established PRRT as standard of care. [S3]
**Radiotheranostic Approvals**	FDA approval of ^{177}Lu-PSMA-617 (Pluvicto) for metastatic castration-resistant prostate cancer	2022	Regulatory validation of PSMA-targeted radionuclide therapy based on overall-survival benefit in the VISION trial; directly relevant to the manuscript's mCRPC scenario [17].
**SBRT / SABR Trial Evidence**	SABR-COMET: overall survival benefit of oligometastatic SABR (OS 41 vs. 28 months; HR 0.57)	2020	Randomized phase II evidence and long-term follow-up support comprehensive SABR in selected oligometastatic patients [14,18].
**SBRT / SABR Trial Evidence**	CURB: randomized phase II evaluation of stereotactic ablative radiotherapy for oligoprogressive cancer	2024	Provides randomized evidence for local ablative therapy in selected oligoprogressive settings while preserving the manuscript's disease- and protocol-specific framing [32].
**Adaptive RT Expansion**	Clinical deployment of MR-Linac (Unity, MRIdian) with online plan adaptation	2018–2022	Enabled daily anatomy-based plan adaptation with real-time MRI guidance; established online ART as feasible standard at high-volume centers. [S2]

**Adaptive RT Expansion**	Prospective data from AURORA and SMART trials supporting MR-guided SBRT with online adaptation	2022–2024	Validated biology-adaptive dose escalation with real-time organ tracking; bridge between conventional ART and state-guided IRBT delivery. [S1]
**Immuno-RT Combinations**	Prospective multicenter evaluation of stereotactic ablative radiotherapy combined with anti-PD-1 therapy in oligoprogressive disease	2021	Supports prospective evaluation of coordinated local and immune therapy while leaving efficacy, sequencing, and patient selection as active research questions [38].
**Immuno-RT Combinations**	Emerging SBRT + ICI combination data (NRG-LU002, KEYNOTE-799, institutional series)	2020–2024	Growing evidence base for spatially targeted radiation reshaping the tumor immune microenvironment in ways relevant to IRBT framework. [S4]
**Precision Dosimetry**	EANM/MIRD quantitative ^{177}Lu SPECT guidance supporting patient-specific dosimetry	2020–2024	Provides a technical basis for quantitative activity and absorbed-dose estimation in ^{177}Lu therapy; clinical implementation remains agent-, protocol-, and jurisdiction-specific [S5].
**Precision Dosimetry**	Ac-225 PSMA-617 investigational IND programs and first prospective dosimetry frameworks	2022–2025	Illustrates extension of molecularly targeted radionuclide therapy to alpha-particle platforms and the need for agent-specific imaging, dosimetry, safety modeling, and prospective validation [58].

References cited in the main manuscript use the final main-manuscript numbering. Sources cited only in Supplementary File 1 use S-prefixed numbers and are listed below. The two numbering systems are independent and should not be combined into ranges.

Supplementary References

References cited in the main manuscript use the final main-manuscript numbering. Sources cited only in Supplementary File 1 use S-prefixed numbers and are listed below. The two numbering systems are independent and should not be combined into ranges.

72. **S1.** Winkel D, Bol GH, Kroon PS, et al. Adaptive radiotherapy: The Elekta Unity MR-linac concept review of clinical implications, current treatment uncertainties and future perspectives. *Radiother Oncol.* 2019;133:55–62. doi: 10.1016/j.radonc.2019.01.022.
73. **S2.** Soukup T, Lamb BW, Arora S, et al. Successful strategies in implementing a multidisciplinary team working in the care of patients with cancer: an overview and synthesis of the available literature. *J Multidiscip Healthc.* 2012;5:49–58. doi: 10.2147/JMDH.S25333.
74. **S3.** Strosberg J, El-Haddad G, Wolin E, et al. Phase 3 Trial of ^{177}Lu-Dotatate for Midgut Neuroendocrine Tumors (NETTER-1). *N Engl J Med.* 2017;376:125–135. doi: 10.1056/NEJMoa1607427.
75. **S4.** Theelen WSME, Peulen HMU, Lalezari F, et al. Effect of Pembrolizumab After Stereotactic Body Radiotherapy vs Pembrolizumab Alone on Tumor Response in Patients With Advanced Non-Small Cell Lung Cancer: Results of the PEMBRO-RT Phase 2 Randomized Clinical Trial. *JAMA Oncol.* 2019;5(9):1276–1282. doi: 10.1001/jamaoncol.2019.1478.
76. **S5.** Ljungberg M, Celler A, Konijnenberg MW, et al. MIRD Pamphlet No. 26: Joint EANM/MIRD Guidelines for Quantitative ^{177}Lu SPECT Applied for Dosimetry of Radiopharmaceutical Therapy. *J Nucl Med.* 2016;57(1):151–162. doi: 10.2967/jnumed.115.159012.
77. **S6.** Ahmed HZ, Nittala MR, Kosalram N, Crosswhite B, Lee AP, Frazier TC, Milner CP, Vijayakumar S. Patients and Families' Participation in Multidisciplinary Tumor Conferences Improves Patient and Family-Focused Cancer Care: Lessons Learned From a Debate on the Role of Radiation Therapy in Primary Mediastinal Non-Hodgkin Lymphoma. *Cureus.* 2023 Feb 6;15(2):e34693. doi: 10.7759/cureus.34693. PMID: 36909112; PMCID: PMC9995141.
78. **S7.** Lindsay HB, Scheurer ME, Allam AK, Lucas BJ, McGovern SL, Chintagumpala M, Paulino AC. Late-onset lymphopenia during radiation is associated with an increased risk of tumor recurrence in newly diagnosed pediatric medulloblastoma. *Pediatr Blood Cancer.* 2024 Jul;71(7):e31022. doi: 10.1002/pbc.31022. PMID: 38644606.
79. **S8.** Vassantachart A, Olch AJ, Jones M, Marques C, Ronckers C, Constine LS, et al. A comprehensive review of 30 years of pediatric clinical trial radiotherapy dose constraints. *Pediatr Blood Cancer.* 2023 May;70(5):e30270. doi: 10.1002/pbc.30270. PMID: 36880707.

End of Supplementary File 1

Supplementary File S2

State-Based Framework Categorization (SBFC) Clinical Worksheet

Integrated Radiobiotherapy (IRBT): A State-Based Framework for Response-Adaptive Combination Oncology

Instructions for Use:

This worksheet operationalizes the SBFC system defined in Section 3.2 of the manuscript as an illustrative research-design template. At each protocol-defined assessment point, investigators may characterize the four variables below and assign a provisional SBFC state for prospective evaluation. The resulting classification may inform candidate modality, treatment intent, reassessment strategy, and safety-monitoring plans, but it does not independently determine clinical management. Numerical thresholds, monitoring intervals, treatment-hold criteria, and stopping rules must be defined and justified separately for each disease, agent, radiation regimen, and study protocol; they are not established clinical practice mandates.

Four Framework Variables (from Section 2):

- **A. Molecular Imaging Phenotype:** Target receptor/tracer expression on PET/SPECT imaging
- **B. Lesion Geometry:** Number, size, and spatial distribution of active lesions
- **C. Systemic Burden & Tempo:** Total disease burden (% stable/progressing); rate of progression
- **D. OAR Constraints:** Organ-at-risk dose tolerance; absolute lymphocyte count (ALC) status

SBFC State Classification Table

The entries below are illustrative trial-design examples. They must not be used as patient-specific treatment recommendations or as universal eligibility, monitoring, dose-hold, escalation, or stopping criteria. Any prospective protocol using this framework must prespecify disease- and agent-specific thresholds, safety oversight, organ-at-risk constraints, laboratory schedules, adverse-event rules, and statistical stopping boundaries with appropriate multidisciplinary and regulatory review.

SBFC State	Clinical Definition	Variable A (Imaging Phenotype)	Variable B (Lesion Geometry)	Variable C (Systemic Burden/ Tempo)	Variable D (OAR/ ALC Constraints)	Treatment Intent	Primary Modality	Monitoring Protocol	Stopping/ Escalation Rules	Evidence Status
**SBFC-0** Baseline Controlled Status	Uniform disease stability or complete response on systemic therapy; no active progression detectable	Negative or stable on functional imaging	No active lesions; complete response or minimal residual	Predominantly stable or responding disease burden with indolent or absent progression; any quantitative definition must be protocol-specific.	OAR normal; ALC within institutional reference range	Observation according to disease-specific standards; no state-based radiation intervention proposed.	None (active surveillance)	Disease- and regimen-specific systemic monitoring and imaging at protocol-defined intervals.	Reclassify if focal progression or other protocol-defined state transition occurs.	[Evidence Status: Illustrative proposed framework category; observation follows disease-specific standards rather than a separately validated SBFC protocol.]
**SBFC-1 / SBFC-OP** — Oligoprogressive Transition	Emergence of a protocol-defined limited number of progressing	Progressing lesions FDG-avid or receptor-positive on interim imaging	Limited active or progressing lesions within a protocol-defined	Predominantly stable or responding systemic disease with low progressi	Hematologic status and organ-at-risk constraints must	Evaluation of local therapy to control focal progression	SBRT to progressing sites, with SABR terminology used when emphasizing	CBC, metabolic testing, toxicity assessment,	Hold, discontinue, or reclassify according to prespecified protocol criteria for	[Evidence Status: Prospective and randomized evidence

	lesions while disease elsewhere remains controlled, creating a potential research window for local therapy intended to control focal progression and possibly prolong benefit from an otherwise effective systemic regimen. Lesion-count criteria must be disease- and protocol-specific.	g; remainder PET-negative or stable	oligoprogressive range; remaining disease controlled or responding.	on tempo; any quantitative burden threshold is illustrative and protocol-specific.	satisfy disease-, agent-, and protocol-specific eligibility criteria; no universal ALC cutoff is established.	and potentially prolong benefit from an otherwise effective systemic regimen; treatment intent, eligibility criteria, and systemic-therapy sequencing must be disease- and protocol-specific.	ng ablative intent; continue or modify systemic therapy according to protocol.	and restaging imaging at agent-, disease-, and protocol-defined intervals.	new progression, hematologic change, non-hematologic toxicity, or emergence of a targetable cross-modality option.	supports local therapy in selected oligoprogressive settings [30–35]; specific lesion-count and burden thresholds remain hypothesis-generating.]
**SBFC-2 / SBFC-TPCI** — Oligometastatic Presentation	Protocol-defined low-volume metastatic disease in which all macroscopically visible sites may be considered for comprehensive local treatment	All sites FDG-avid or receptor-positive and targetable; no occult dissemination on whole-body imaging	A limited number of discrete metastatic sites within disease- and protocol-defined eligibility criteria; no immediate	Low-to-moderate total burden; stable or slowly progressing tempo	Hematologic status and organ-at-risk constraints must meet disease-, regimen-, and protocol-specific eligibil	Evaluation of comprehensive local treatment to visible disease with disease-control or potentially curative intent only when	SBRT to eligible metastatic sites, with SABR terminology used when emphasizing ablative intent, with or without systemic therapy; TRT combinations	CBC, metabolic testing, toxicity assessment, and restaging imaging at disease-, regimen-, and	Reassess eligibility and treatment intent according to prespecified protocol criteria for new disease sites, hematologic change, organ-at-risk constraints, or	[Evidence Status: Randomized phase II data support comprehensive local therapy in selected oligometastatic settings [14,18,30,42,44–

	within disease-specific standards or research protocols.		visceral crisis.		ity criteria; no universal ALC cutoff is established.	supported by disease-specific standards or explicitly defined in a prospective protocol.	remain investigational and protocol-specific.	protocol-defined intervals.	unexpected toxicity.	45]; integration of TRT with multisite SBRT remains investigational.]
**SBFC-3 / SBFC-SyBP** Symptomatic Bulky Progression / Palliative Potential	Bulky, symptomatic, or organ-threatening progression with a large hypoxic or necrotic mass that may prompt urgent evaluation of a local palliative intervention.	Heterogeneous uptake; large hypoxic core may be FDG-negative or PSMA-poor	Bulky single or regional mass; not amenable to standard fractionated focal ablation	High local burden with mechanical threat; systemic disease variable	ALC may be compromised; OAR constraints require specialized planning (SFRT-specific)	Palliative intent (symptom relief, local tumor control, decompression)	SFRT/GRID/Lattice radiotherapy ± systemic therapy; TRT if receptor-positive systemic component	Symptom assessment, CBC with ALC, metabolic testing, and imaging at disease-, technique-, and protocol-defined intervals.	Hold, modify, or discontinue according to protocol-defined organ-function, hematologic, toxicity, symptom, and treatment-goal criteria.	[Evidence Status: Institutional series and systematic reviews support palliative SFRT/Lattice use in selected bulky tumors, but evidence is heterogeneous and does not establish universal schedules or stopping criteria [26,43,46–47].]
**SBFC-4 / SBFC-AsyP** Asymptomatic	Rapid systemic progression; high metabolic	High-volume diffuse TRT-targeta	Diffuse multisite or visceral	High and rapidly progressing systemic	Hematologic status, prior	Palliative intent (systemic	Carefully selected palliative or sub-	CBC with ALC, organ	Hold, modify, discontinue, or	[Evidence Status: Radiatio

High-Burden Progression / Palliative Potential	/receptor-positive volume across multiple visceral organs; no immediate local crisis; immune-preservation paramount	ble uptake (e.g., PSMA, SSTR, DOTATATE) on whole-body imaging	disease not suitable for comprehensive focal ablation; any lesion-count boundary is illustrative and protocol-specific.	burden; high tempo	therapy, organ function, and organ-at-risk constraints guide whether any local radiation is appropriate; no universal ALC or dose threshold is established.	disease control, immune preservation, ALC safety)	ablative EBRT may be considered for dominant symptomatic sites; systemic therapy or TRT selection remains disease-, target-, and protocol-specific.	-function testing, toxicity assessment, and imaging at agent-, disease-, and protocol-defined intervals.	switch treatment according to prespecified protocol criteria for hematologic change, organ toxicity, disease progression, or inadequate response.	n-induced lymphopenia literature supports attention to irradiated volume and hematologic reserve [28–29], but specific ALC, dose, and combined-modality thresholds remain investigational.]
**SBFC-5 / SBFC-TINV** Investigational Cross-Modality Transition	Complex biological scenario with active investigational options; concurrent or sequential combination of systemic TRT and high-dose focal EBRT consolidation within prospective clinical trial	Protocol-defined target expression on validated imaging or biomarkers, with one or more dominant lesions potentially suitable for	Systemic target-expressing disease with protocol-defined focal dominant or resistant lesion(s) considered for EBRT consolidation.	Variable; typically moderate systemic burden with identified focal resistant/dominant clone	Hematologic status, organ function, prior therapy, and cumulative dosimetry must meet agent- and protocol-specific eligibility	Investigational intent; prospective evaluation should occur within an appropriately reviewed clinical trial or research protocol.	Protocol-defined sequential or concurrent TRT with focal SBRT, using SABR terminology when emphasizing ablative intent, evaluated within an appropriately reviewed prospectiv	CBC, metabolic and organ-function testing, toxicity assessment, imaging, and dosimetry according to the	Apply prespecified protocol stopping, dose-modification, and safety-review rules for hematologic toxicity, organ toxicity, unexpected adverse events, or disease progression.	[Evidence Status: Investigational. Reviews, radiobiological analyses, and early clinical studies support prospective evaluation of combined TRT and

		focal EBRT; no universal uptake threshold is established.			criteria before enrollment.		e trial or research framework.	approved protocol, with independent safety oversight where required.		focal EBRT, but sequencing, safety, and efficacy remain unvalidated [15,48–53].]

Notes for Clinical Trial Adaptation

1. **Threshold Provisionality:** All numerical cutoffs shown in the worksheet—including lesion-count, systemic-burden, ALC, monitoring, and treatment-hold examples—are illustrative trial-design parameters rather than mandated clinical criteria. Evidence supports attention to disease extent, irradiated volume, and hematologic reserve, but it does not validate universal numerical thresholds [1,16,28–29]. Prospective multi-institutional protocols must define and justify their own criteria.

2. **TRT Agent Specificity:** Monitoring schedules, stopping rules, imaging requirements, dosimetry methods, and patient-selection criteria are not uniformly applicable across targeted radionuclide therapy agents. Target biology, physical half-life, biodistribution, organ at risk, prior therapy, and hematologic tolerance vary by agent and disease setting [15,17,50–53,59,66]. Each protocol must specify agent-specific safety and dosimetry parameters.

3. **Reassessment Nodes:** SBFC state assignment is not permanent. Formal reassessment at each prespecified treatment node (as per Section 2 adaptive loop) may reclassify a patient to a higher or lower SBFC state, triggering protocol-specified escalation, consolidation, switch, or de-escalation.

4. **Cross-Reference to Main Manuscript:**

- **Four-variable framework and adaptive decision loop: Sections 2–2.1**
- Implications for medical oncology: Section 2.2
- SBFC definitions and provisional thresholds: Sections 3.2–3.3
- Disease-specific scenarios: Sections 4.1–4.4

References

References cited in the main manuscript use the final main-manuscript numbering. Supplementary File 2 has no supplementary-only references; therefore, all bracketed references in this file correspond to the numbered main-manuscript reference list.

End of Supplementary File 2

Supplementary File S3

Cumulative TRT–EBRT Dosimetry Calculation Framework & Sensitivity Protocol

Manuscript: Integrated Radiobiotherapy: A State-Based Framework for Response-Adaptive Combination Oncology

Journal: *Current Oncology*

Cross-reference: Main text Section 5.2 (Cumulative Dosimetry & Radiobiological Modeling)

Overview

This supplementary file presents mathematical derivations, parameter definitions, representative reference values, and sensitivity analyses supporting the cumulative dosimetry discussion in Section 5.2 of the main manuscript. It is intended as a research-design and reporting framework for radiation oncologists, medical physicists, and clinical trialists evaluating biologically modeled doses in combined External Beam Radiotherapy (EBRT) and Targeted Radionuclide Therapy (TRT) sequences. It is not a validated clinical calculator, treatment-planning protocol, or substitute for patient-specific dosimetry.

The formulas presented here use the linear-quadratic framework described by Fowler [61] and the combined EBRT–TRT dosimetry approach described by Bodey, Flux, and Evans [52]. Parameter values are illustrative. Prospective clinical application requires quantitative patient-specific imaging and dosimetry, explicit reporting of model assumptions and uncertainty, and voxel-level reconstruction when technically feasible and appropriate to the study question [15,52–53,59–60,63].

S3.1 The Linear-Quadratic Model: Biologically Effective Dose (BED)

S3.1.1 Foundational Formula

The linear-quadratic model relates cell survival after irradiation to linear and quadratic components of radiation effect. For conventionally fractionated radiotherapy, biologically effective dose is expressed as follows [61]:

$$\mathbf{BED = D \times (1 + d / (\alpha/\beta))}$$

where:

- **D** = total absorbed physical dose (Gy)
- **d** = dose per fraction (Gy)
- **α/β** = tissue-specific LQ radiosensitivity ratio (Gy); characterizes the curvature of the cell-survival curve

The BED provides a common radiobiological currency that accounts for fraction-size effects and allows comparison across fractionation schedules.

S3.1.2 Equivalent Dose in 2 Gy Fractions (EQD2)

For fractionated radiotherapy schedules, EQD2 expresses a modeled biologically equivalent dose in 2 Gy fractions. Application to protracted TRT exposure requires a separately specified dose-rate and repair model rather than direct use of the fractionated-dose expression:

$$\mathbf{EQD2 = D \times (d + \alpha/\beta) / (2 + \alpha/\beta)}$$

Equivalently:

$$\mathbf{EQD2 = BED / (1 + 2 / (\alpha/\beta))}$$

EQD2 applies to fractionated schedules; its use for protracted targeted radionuclide therapy (TRT) requires explicit assumptions about dose rate, repair, and model applicability. In combined EBRT–TRT research, it may provide a reporting framework only when the underlying dose-rate, repair, spatial-distribution, and model assumptions are explicitly stated; direct arithmetic summation across modalities should not be treated as validated without appropriate radiobiological modeling.

Derivation note: EQD2 follows from setting BED equal for two schedules (D, d) and (EQD2, 2 Gy), then solving for EQD2. The formula simplifies to the expression above [61,S12].

S3.2 Modified BED Formula for Targeted Radionuclide Therapy (TRT)

S3.2.1 Rationale for Modification

Unlike fractionated EBRT — which delivers discrete, high-dose-rate pulses — TRT irradiates tissues at a continuously decaying, low dose rate as the administered radionuclide undergoes physical decay and biological clearance. This prolonged, low-dose-rate (LDR) exposure allows substantial sub-lethal damage repair between "dose events," substantially altering the radiobiological effect relative to what would be predicted by the standard fractionated BED formula [S9, S13].

The Dale formula is a foundational modeling approach for monoexponential protracted irradiation rather than the standard quantitative tool.

S3.2.2 Dale (1985) Formula for Monoexponential Dose Rate Decay

In the monoexponential model, the effective decay constant incorporates both physical decay and biological clearance.

Total absorbed dose:

$$D\_TRT = \dot{R}_0 / \lambda$$

BED for TRT:

$$BED\_TRT = D\_TRT \times [1 + \dot{R}_0 / ((\alpha/\beta) \times (\mu + \lambda))]$$

Equivalently (substituting $\dot{R}_0 = D\_TRT \times \lambda$):

$$BED\_TRT = D\_TRT \times [1 + (\lambda / (\mu + \lambda)) \times (D\_TRT / (\alpha/\beta))]$$

$$BED\_TRT = D\_TRT + (g \times D\_TRT^2) / (\alpha/\beta)$$

The parameter g ranges from 0 to 1. Values near 1 correspond to little repair during short or effectively instantaneous delivery and preservation of the quadratic contribution, whereas values near 0 correspond to extensive repair during protracted delivery and marked reduction of the quadratic contribution. For clinically used radionuclides with long effective half-lives and tissues with rapid sublethal-damage repair ($T½\_repair$ = 1–3 h):

- $\dot{\mathbf{R}}_0$ = initial absorbed dose rate at the tissue of interest (Gy/h); determined by patient-specific dosimetry
- **λ = effective decay constant of the tissue time–activity curve (h^{-1}) = ln 2 / T½_effective; when physical decay and monoexponential biological clearance are independent, λ = λphysical + λbiological.**
- **μ** = sub-lethal damage repair rate constant (h^{-1}) = ln 2 / T½_repair
- **T½_repair** = repair half-time of the tissue of interest (h); see Table S3.2
- **For the Lea-Catcheside factor, g near 1 corresponds to little repair or short delivery, whereas g near 0 corresponds to extensive repair during protracted delivery.**

S3.2.3 Physical Interpretation of the Lea-Catcheside Factor (g)

The parameter **g** ranges from 0 to 1. Values near 1 correspond to short or effectively instantaneous delivery with little repair during irradiation and preservation of the quadratic contribution. Values near 0 correspond to prolonged low-dose-rate delivery with extensive sublethal-damage repair and marked reduction of the quadratic contribution. For clinically used radionuclides with long physical half-lives (days to weeks) and tissues with rapid sublethal repair (T½_repair = 1–3 h):

Since λ ($\approx 10^{-3}$ h^{-1}) << μ ($\approx$ 0.2–0.7 h^{-1}):
$$\mathbf{g = \lambda / (\mu + \lambda) \approx \lambda / \mu \approx 0.005 - 0.02}$$
This small value of g confirms that TRT behaves as near-pure LDR irradiation: almost all sub-lethal damage is repaired during delivery, and the BED is only modestly above the physical absorbed dose. The clinical implication is that TRT contributes lower biologically effective dose per unit absorbed dose than hypofractionated EBRT but delivers dose continuously throughout the entire treatment period.

Representative computed g values are provided in Table S3.3.

S3.2.4 EQD2 from TRT

After computing BED_TRT, the EQD2 from TRT is:

EQD2_TRT = BED_TRT / (1 + 2 / (α/β))

S3.3 Cumulative EBRT + TRT Dosimetry

S3.3.1 BED and EQD2 Summation

When EBRT and TRT deliver dose to the same tissue, their modeled BED contributions may be summed as a first-order approximation only when compatible α/β assumptions, dose-rate and repair models, spatial mapping, temporal sequencing, and uncertainty treatment are used.

BED_total = BED_EBRT + BED_TRT
EQD2_total = BED_total / (1 + 2 / (α/β))

Key requirement: Both BED components must use the same α/β value, reflecting the same tissue type. Mixing α/β assumptions across components will yield a non-interpretable combined EQD2.

S3.3.2 Temporal Sequencing Consideration

If TRT and EBRT are not delivered simultaneously, their combined biological effect depends on repair kinetics, dose-rate history, spatial overlap, and the interval between exposures. BED summation is therefore a modeling approximation that requires compatible alpha/beta assumptions, explicit dose-rate and repair models, spatial mapping, temporal sequencing, and uncertainty analysis. When short intervals or overlapping biological effects are clinically relevant, an explicitly specified temporal-repair model should be used and justified; no universal correction factor is established. Detailed modeling considerations are discussed in references S9 and S13.

S3.4 Parameter Reference Tables

Table S3.1: Representative α/β Ratios by Tissue and Tumor Type

Tissue / Tumor Type	α/β Ratio (Gy)	Clinical Note
Late-responding normal tissues (spinal cord, rectum, bladder)	1.5–3.0 (commonly **2.0**)	Illustrative late-responding normal-tissue value; actual OAR assumptions must be tissue-, endpoint-, and protocol-specific.
Prostate cancer (slow-growing)	1.5–2.0	Supports hypofractionated and SBRT regimens for prostate cancer

Breast cancer	3.0–4.0	Supports moderate hypofractionation (e.g., 40 Gy/15 fx)
Lung parenchyma (late)	2.5–3.5 (commonly **3.0**)	Mixed fast and slow repair compartments
Kidneys (late, critical OAR for ^{177}Lu therapy)	2.0–2.5	Key constraint for ^{177}Lu-PSMA and ^{177}Lu-DOTATATE dosimetry
Liver (late)	1.5–2.5 (commonly **2.0**)	Illustrative value requiring tissue-, endpoint-, agent-, and protocol-specific justification for TRT liver dosimetry
Bone marrow / hematopoietic stem cells	8–12	Early-responding compartment; less restrictive in late-effect modeling
Neuroendocrine tumors (NETs)	8–10	Illustrative value requiring tissue-, endpoint-, agent-, and protocol-specific justification; used in ^{177}Lu-DOTATATE treatment planning
Non-small cell lung cancer (NSCLC)	8–10	Illustrative value requiring tissue-, endpoint-, agent-, and protocol-specific justification; supports SBRT ablative dosing
Pediatric normal tissues	3–5 (uncertain, age-dependent)	Conservative; evidence limited; individual dosimetry recommended [61–62]

All values are approximate population-level estimates based on radiobiological modeling and clinical trial data. Individual variation exists. Sources: [61,S11,S12].

Table S3.2: Tissue Repair Half-Times and Rate Constants (μ) for Late-Responding Normal Tissues

Tissue Type	Repair Half-Time T½_repair (h)	Repair Rate μ (h^{-1})	Primary OAR Relevance
CNS / Spinal cord	1.5 (range: 1.0–2.0)	**0.462**	Critical OAR for EBRT; slow-repair correction critical for SBRT
Lung parenchyma	2.0 (range: 1.5–3.0)	**0.347**	Late lung fibrosis endpoint
Liver	1.5 (range: 1.0–2.5)	**0.462**	Hepatic TRT dosimetry; SBRT stereotactic liver plans
Kidneys	2.5 (range: 1.5–4.0)	**0.277**	Critical late-effect OAR for ^{177}Lu-PSMA and ^{177}Lu-DOTATATE
Rectum / Bowel (late)	1.5 (range: 1.0–2.5)	**0.462**	Illustrative value requiring tissue-, endpoint-, agent-, and protocol-specific justification
Bone marrow (late)	1.0 (range: 0.5–1.5)	**0.693**	Hematologic toxicity endpoint for systemic TRT

Default (late tissue, if unknown)	**1.5**	**0.462**	Illustrative value requiring tissue-, endpoint-, agent-, and protocol-specific justification in absence of tissue-specific data

Formula: $\mu = \ln 2 / T½\_repair$. Values from references [S9, S11, S12].

Table S3.3: Physical Decay Constants and Illustrative Lea–Catcheside Factors for ^{177}Lu and ^{223}Ra

Radionuclide	Physical T½ (days)	Physical T½ (hours)	λ (h^{-1})	g = λ/(μ + λ)†	Clinical Applications
^{177}Lutetium (^{177}Lu)	6.647	159.5	**0.00434**	**≈ 0.0093**	^{177}Lu-DOTATATE (NETs); ^{177}Lu-PSMA-617 (mCRPC)
^{223}Radium (^{223}Ra)	11.43	274.3	**0.00253**	**≈ 0.0054**	Radium-223 dichloride (bone metastases, mCRPC)

†Values of g were calculated using an illustrative repair half-time of 1.5 h ($\mu = 0.462\ h^{-1}$). Values near 1 indicate little repair during short delivery, whereas values near 0 indicate extensive repair during protracted delivery.

^{177}Lu: $g = 0.00434 / (0.462 + 0.00434) \approx 0.0093$; ^{223}Ra: $g = 0.00253 / (0.462 + 0.00253) \approx 0.0054$.

Formula: $\lambda = \ln 2 / T½\_physical$. Sources: [S10, S13, S14].

Interpretation: The very small g values (< 0.01) for both radionuclides confirm near-pure LDR behavior: sub-lethal repair is rapid relative to the rate of dose delivery. Summation of BED contributions is a modeling approximation that requires compatible alpha/beta assumptions, dose-rate and repair modeling, spatial mapping, temporal sequencing, and uncertainty analysis.

S3.5 Sensitivity Analysis: EQD2 Variation Across α/β and SBRT Fraction Size

The sensitivity analysis below evaluates how changing the assumed α/β ratio from 2 Gy to 4 Gy alters calculated EQD2 across representative hypofractionated and SBRT schedules; the resulting differences are specific to the modeled schedules and assumptions.

Formula applied:

$$EQD2 = D \times (d + \alpha/\beta) / (2 + \alpha/\beta)$$

Table S3.4: SBRT Sensitivity Analysis — EQD2 Variation by α/β and Fraction Scheme

SBRT Regimen	Total D (Gy)	d per Fraction (Gy)	BED (α/β = 2)	EQD2 (α/β = 2)	BED (α/β = 4)	EQD2 (α/β = 4)	EQD2 Difference (%)
50 Gy / 5 fx	50	10.0	300 Gy	**150.0 Gy**	175 Gy	**116.7 Gy**	**22.2%**
60 Gy / 5 fx	60	12.0	420 Gy	**210.0 Gy**	240 Gy	**160.0 Gy**	**23.8%**
48 Gy / 4 fx	48	12.0	336 Gy	**168.0 Gy**	192 Gy	**128.0 Gy**	**23.8%**
54 Gy / 3 fx	54	18.0	540 Gy	**270.0 Gy**	297 Gy	**198.0 Gy**	**26.7%**
60 Gy / 3 fx	60	20.0	660 Gy	**330.0 Gy**	360 Gy	**240.0 Gy**	**27.3%**
Illustrative 30 Gy in 5 fractions†	30	6.0	120 Gy	**60.0 Gy**	75 Gy	**50.0 Gy**	**16.7%**
45 Gy / 3 fx	45	15.0	382.5 Gy	**191.3 Gy**	213.75 Gy	**142.5 Gy**	**25.5%**

†30 Gy in 5 fractions is included as an illustrative hypofractionated regimen; its classification and clinical intent are context-dependent. Its smaller EQD2 difference reflects the more moderate fraction size relative to the assumed α/β ratios.

Summary: Across clinically used SBRT regimens, the EQD2 difference between α/β = 2 Gy and α/β = 4 Gy ranges from approximately **17% to 27%**, with higher-dose-per-fraction schedules producing the largest divergence. The 50 Gy/5 fx regimen — a widely used SBRT schedule for oligometastatic disease — yields a **22.2% difference**, consistent with the main text statement [62]. **Across all modeled schedules, changing α/β from 2 Gy to 4 Gy changes EQD2 by approximately 17–27%; among the regimens explicitly presented as SBRT schedules, the difference is approximately 22–27%. Combined EBRT–TRT studies should prespecify α/β assumptions, quantify their influence in sensitivity analyses, and report the resulting uncertainty.**

S3.6 Worked Clinical Example: Combined ^{177}Lu-PSMA and SBRT Cumulative Dosimetry (Illustrative Only)

The following example is provided for conceptual illustration only and does not constitute a clinical dose prescription. Clinical application requires patient-specific dosimetry based on appropriate quantitative imaging and uncertainty assessment [59,63,S10,S14].

Scenario: A patient with metastatic castration-resistant prostate cancer (mCRPC) receiving combined ^{177}Lu-PSMA therapy and focal SBRT consolidation to a bone metastasis.

Given values (representative, not patient-specific):

- EBRT component: SBRT 50 Gy / 5 fractions (10 Gy/fx) to bone lesion
- TRT component: ^{177}Lu-PSMA, total absorbed dose to adjacent normal bone = 10 Gy (D_TRT)
- $\dot{R}_0$ = D_TRT × λeffective = 10 Gy × 0.00434 h^{-1} = 0.0434 Gy/h under the illustrative no-biological-clearance assumption.
- α/β for late bone / bone marrow stroma: 2.0 Gy (conservative)
- Repair half-time T½_repair = 1.5 h → μ = 0.462 h^{-1}
- For illustration only, λeffective is assumed equal to the ^{177}Lu physical decay constant, 0.00434 h^{-1}, with no additional biological clearance, giving g ≈ 0.0093.

Step 1: BED from EBRT

BED_EBRT = 50 × (1 + 10/2) = 50 × 6 = **300 Gy**
EQD2_EBRT = 300 / (1 + 2/2) = 300/2 = **150 Gy**

Step 2: BED from TRT

BED_TRT = D_TRT + (g × D_TRT²) / (α/β)
= 10 + (0.0093 × 100) / 2.0
= 10 + 0.465
= **10.5 Gy**
EQD2_TRT = 10.5 / 2 = **5.2 Gy**

Step 3: Cumulative EQD2

EQD2_total = EQD2_EBRT + EQD2_TRT = 150 + 5.2 = **155.2 Gy**

Interpretation: In this illustrative scenario, the TRT component adds a modest but non-negligible biologically effective dose (~5 EQD2 Gy) beyond the SBRT contribution. The near-zero g factor for ^{177}Lu confirms that TRT behaves predominantly as LDR irradiation; at modest absorbed doses (< 20 Gy), the TRT BED increment above D_TRT is small. Higher TRT absorbed doses (50–100 Gy, as may occur in tumor targets) would yield larger BED contributions from the quadratic term.

S3.7 Clinical Guidance and Limitations

1. **Patient-specific dosimetry is essential.** The representative parameter values in Tables S3.1–S3.3 are population-level estimates. Individual variation in tumor uptake, renal clearance, organ volume, and radiosensitivity may result in substantial deviations from tabulated values. Patient-specific dosimetry using quantitative SPECT/CT or PET/CT is recommended when technically feasible and appropriate [59,63,S10,S14].

2. **Single α/β assumption per tissue.** The LQ model assumes a single, homogeneous α/β ratio for each tissue compartment. In reality, tissues contain mixed cell populations with different radiosensitivities. Voxel-level dosimetry may be useful depending on technical feasibility and the study question [63].

3. Biologically effective dose additivity is a first-order approximation. Summation of BED contributions requires compatible α/β assumptions, dose-rate and repair modeling, spatial mapping, temporal sequencing, and uncertainty analysis. Additivity may not hold when temporal overlap, reoxygenation, or treatment-related biological effects alter the combined response [S13].

4. **Repair kinetics may change with prior treatment.** In patients who have received prior cytotoxic chemotherapy, targeted agents, or immunotherapy, tissue repair kinetics (T½_repair) and radiosensitivity (α/β) may be altered. Default parameter values from Table S3.2 should be used with caution in heavily pre-treated patients.

5. ^{177}Lu multi-cycle dosimetry. Many ^{177}Lu regimens use multiple cycles separated by protocol-defined intervals. Residual interaction depends on repair kinetics, the interval between exposures, dose-rate history, and model assumptions. When a common tissue-specific α/β value and compatible dose-rate model are used, cycle-specific BED contributions may be summed as a first-order approximation and the cumulative BED then converted once to EQD2; direct summation of independently rounded EQD2 values should not be presented as a separately validated biological model.

6. **^{223}Ra bone dosimetry caveats.** Radium-223 emits short-range alpha particles (~0.1 mm tissue range) targeting bone surface and marrow. Dose is highly heterogeneous at the microscopic level. Mean absorbed dose estimates underrepresent focal hotspots at trabecular bone surfaces. The LQ/BED framework provides only a macroscopic dosimetric approximation for ^{223}Ra and should be interpreted with particular caution [S14].

7. SBRT α/β uncertainty propagates nonlinearly. As shown in Table S3.4, modeled EQD2 is sensitive to the assumed α/β value. When tissue-specific evidence is uncertain, analyses should report results across a justified range of α/β values; selection of an OAR constraint and decision rule must follow the applicable clinical protocol rather than a universal higher-EQD2 mandate.

Supplementary References

Main-manuscript references use final numbering. References S9–S14 are supplementary-only and are listed below. The two numbering systems are independent and should not be combined into ranges.

80. **S9.** Dale RG. The application of the linear-quadratic dose-effect equation to fractionated and protracted radiotherapy. *Br J Radiol.* 1985;58(690):515–528. doi:10.1259/0007-1285-58-690-515. PMID: 4063711.

(Original derivation of the modified BED formula for protracted and continuously decaying dose-rate irradiation; foundational reference for TRT radiobiology.)

81. **S10.** Lassmann M, Eberlein U. The relevance of dosimetry in precision medicine. *J Nucl Med.* 2018;59(Suppl 1):1S–3S. doi:10.2967/jnumed.118.210773.
(Clinical implementation of personalized TRT dosimetry; review of quantitative SPECT/CT and PET/CT-based absorbed dose measurement.)

82. **S11.** Strigari L, Konijnenberg M, Chiesa C, et al. The evidence base for the use of internal dosimetry in the clinical practice of molecular radiotherapy. *Eur J Nucl Med Mol Imaging.* 2014;41(10):1976–1988. doi:10.1007/s00259-014-2824-5.
(Evidence review for clinical TRT dosimetry; tissue-specific α/β and repair kinetics for combined EBRT–TRT calculations.)

83. **S12.** Joiner MC, van der Kogel AJ, eds. *Basic Clinical Radiobiology.* 4th ed. London: Hodder Arnold; 2009. ISBN: 978-0340929667.
(Standard reference text for LQ model parameters, tissue α/β values, repair kinetics, and fractionation radiobiology.)

84. **S13.** O'Donoghue JA. The relevance of the linear-quadratic model to radionuclide therapy. *Cancer Biother Radiopharm.* 2000;15(1):13–17. doi:10.1089/cbr.2000.15.13.
(Analysis of the applicability and limitations of linear-quadratic modeling in radionuclide therapy, including dose-rate and repair effects.)

85. **S14.** Sgouros G, Bodei L, McDevitt MR, Nedrow JR. Radiopharmaceutical therapy in cancer: clinical advances and challenges. *Nat Rev Drug Discov.* 2020;19(9):589–608. doi:10.1038/s41573-020-0073-9.
(Comprehensive review of ^{177}Lu and ^{223}Ra clinical dosimetry, dose-response relationships, and absorbed dose benchmarks across tumor types and normal tissues.)

End of Supplementary File 3

Supplementary File 3 of 4 | Document version: September 2026 | For questions regarding dosimetric calculations, please contact the corresponding author.

Supplementary File S4: International Prospective Registry Protocol Outline

Global Integrated Radiobiotherapy & Radiobiomedicine Registry (G-IRBT Registry)

A Multi-Center Prospective Observational Study of State-Based Response-Adaptive Radiation Sequencing

Status: Proposed Draft Template — For Scientific and Regulatory Discussion

Section 1: Registry Identity and Title

Full Title: Global Integrated Radiobiotherapy & Radiobiomedicine Registry (G-IRBT Registry): A Multi-Center Prospective Observational Study of State-Based Response-Adaptive Radiation Sequencing in Oncology

Short Title / Acronym: G-IRBT Registry

Registry Type: Multi-center, international, prospective observational cohort registry

Version: Protocol Outline v1.0 (Draft Template)

Date: September 2026

Section 2: Background and Scientific Rationale

2.1 The Problem Being Addressed

Contemporary oncology practice relies on sequential, modality-specific handoffs between radiation oncology, medical oncology, and nuclear medicine. These handoffs are structured around anatomic staging at baseline rather than dynamic assessment of treatment response. As a consequence, opportunities to integrate targeted radionuclide therapy (TRT), external-beam radiotherapy (EBRT), and systemic biotherapy in response-adapted sequences are routinely missed, and the evidence base for such integration remains observational and fragmented.

The Integrated Radiobiotherapy framework proposes a state-based approach for prospective study of cross-modality treatment sequencing. SBFC categories are intended as provisional research

constructs for longitudinal classification and hypothesis generation; they do not independently determine treatment, replace established staging, or prescribe clinical management.

2.2 The Evidence Gap

No prospective registry currently captures longitudinal outcomes in patients managed according to state-based, response-adaptive radiation sequencing principles. Existing registries for SBRT, TRT, and immunotherapy are modality-specific and do not capture cross-modality integration decisions, SBFC state transitions, or the clinical rationale for sequencing choices at individual decision nodes. This represents a fundamental gap in the evidence infrastructure needed to validate or refute the IRBT framework in real-world practice.

2.3 Justification for a Registry Approach

A prospective registry — rather than a randomized controlled trial — is the appropriate initial study design for several reasons:

1. The heterogeneity of eligible disease presentations (NSCLC, mCRPC, HER2+ MBC, MSS CRC, and others) and intervention combinations makes standardized randomization premature in the absence of established state-based treatment protocols;
2. Real-world practice data are needed to define the natural frequency of SBFC state transitions, to estimate effect sizes for future trial design, and to identify the patient subgroups most likely to benefit from integration strategies;
3. A registry format permits broad international participation, including institutions that are not yet equipped to conduct randomized IRBT trials but can contribute to the foundational evidence base through standardized longitudinal data collection.

Section 3: Objectives

3.1 Primary Objective

To evaluate the feasibility, completeness, and reproducibility of prospective longitudinal data collection using provisional SBFC classifications, including the frequency and timing of state transitions, cross-modality treatment sequences, reassessment practices, and safety outcomes across participating centers.

3.2 Secondary Objectives

1. To characterize the frequency, timing, and clinical triggers of SBFC state transitions (SBFC-0 → **SBFC-OP** / SBFC-TPCI / SBFC-TINV) in real-world multi-center practice;

2. To describe rates and severity of treatment-related toxicity, with particular attention to Grade ≥3 adverse events and severe radiation-induced lymphopenia (ALC < 500/μL);

3. To evaluate longitudinal patient-reported quality of life (QoL) outcomes across SBFC states;

4. To document cumulative TRT–EBRT dosimetric parameters and assess their association with toxicity outcomes;

5. To identify practice variation in state-based adaptation decisions across participating international centers.

3.3 Exploratory Objectives

1. To generate hypotheses regarding optimal sequencing intervals and dose parameters for future prospective interventional trials;

2. To assess the feasibility and inter-rater reliability of SBFC state assignment across clinicians and institutions;

3. To characterize institutional infrastructure requirements (imaging, physics co-planning, MDT structure) associated with successful IRBT implementation.

Section 4: Study Design

Design: Multi-center, prospective, longitudinal observational cohort registry with concurrent comparison cohort (standard-of-care sequential specialty handoff) at participating institutions.

Setting: Academic and community oncology centers with active radiation oncology, medical oncology, and (where available) nuclear medicine/theranostics programs. International participation is a design priority.

Duration: Minimum 5-year enrollment period with minimum 3-year follow-up per patient; total registry duration minimum 8 years.

Cohort Structure:

Cohort	Definition
IRBT Cohort	Patients whose multidisciplinary care generates sufficient data to assign a provisional SBFC category retrospectively or prospectively for observational analysis. SBFC assignment is recorded for research purposes and does not determine treatment.
Comparison Cohort	Concurrent eligible patients at participating institutions whose care is documented without a formal SBFC classification. Any comparative analyses are exploratory and require prespecified adjustment for measured confounding, selection bias, immortal-time bias, and institutional practice variation.

Pilot Enrollment Target: The pilot enrollment target is intended to assess multicenter feasibility, data completeness, classification reproducibility, event rates, and the precision of descriptive estimates. It is not powered for definitive comparative-effectiveness or superiority testing. A formal statistical analysis plan should prespecify feasibility thresholds, expected precision, missing-data handling, and criteria for revising the enrollment target after the planned interim assessment.

Section 5: Eligibility Criteria

5.1 Inclusion Criteria

1. Adult patients (age ≥18 years) with histologically or cytologically confirmed solid malignancy;

2. Disease state meeting criteria for at least one SBFC category (SBFC-1 through SBFC-5) at time of enrollment based on imaging and clinical assessment;

3. Planned or ongoing treatment involving at least one radiation-based modality (EBRT, SBRT/SABR, or TRT) in combination or sequence with at least one systemic agent (immunotherapy, targeted therapy, hormone therapy, or chemotherapy);

4. Ability to provide written informed consent;

5. Availability of baseline cross-sectional imaging (CT, PET/CT, or MRI) within 8 weeks prior to enrollment.

5.2 Exclusion Criteria

1. SBFC-0 status (stable, non-progressing disease) without active cross-modality intervention;

2. Radiation therapy administered as sole modality without concurrent or planned systemic therapy integration;

3. Life expectancy < 3 months at time of enrollment;

4. Participation in a blinded randomized controlled trial in which IRBT-relevant outcomes cannot be independently captured;

5. Inability or unwillingness to complete patient-reported outcome measures at scheduled intervals.

Section 6: SBFC-Based Enrollment and State Assignment

6.1 SBFC Assignment Protocol

Each enrolled patient may receive a provisional SBFC state assignment at enrollment and at each defined clinical reassessment point (see Section 7). SBFC assignment is recorded by the treating multidisciplinary team for observational research using the standardized provisional criteria in Supplementary File 2; it does not determine treatment or replace disease-specific standards and multidisciplinary judgment.

SBFC State	Clinical Definition (Summary)
SBFC-0	Baseline controlled status — uniform stability or response across all disease sites
SBFC-OP (SBFC-1)	Oligoprogressive transition—protocol-defined limited focal progression while disease elsewhere remains controlled.
SBFC-TPCI (SBFC-2)	Oligometastatic presentation—protocol-defined low-volume metastatic disease considered for comprehensive local treatment within disease-specific standards or research protocols.
SBFC-TINV (SBFC-5)	Investigational cross-modality transition—complex scenarios involving proposed TRT–EBRT or biomarker-guided combinations.
SBFC-3 (SBFC-SyBP)	Symptomatic bulky progression—palliative research setting.
SBFC-4 (SBFC-AsyP)	Asymptomatic high-burden progression—systemic-therapy-priority setting.
SBFC-CNS	CNS-dominant progression despite extracranial disease control.

Full SBFC scoring criteria, worksheets, and implementation guidance are provided in **Supplementary File 2** of the IRBT framework document.

6.2 State Transition Documentation

Every SBFC state transition must be documented at the time it occurs, including: (a) the clinical trigger for reassignment (imaging, PSA/biomarker, symptom assessment); (b) the treatment decision made at each transition node; and (c) the responsible clinician and institutional site. This documentation constitutes the primary registry dataset.

Section 7: Data Collection Schema

7.1 Data Collection Nodes

Data collection is structured around SBFC transition nodes rather than fixed calendar time points, in addition to scheduled interval assessments:

Node	Timing
Enrollment/Baseline	At SBFC state assignment, prior to treatment initiation
Transition Node	At every formal SBFC state reassignment
Post-Intervention Assessment	4–8 weeks after each major radiation intervention

Interval Follow-Up	Every 12 weeks (clinical/labs); every 24 weeks (imaging)
Annual Comprehensive Review	Annually from enrollment
End of Active Treatment	At conclusion of indexed treatment course
Long-Term Follow-Up	Annually for minimum 3 years post-enrollment

7.2 Data Elements

Clinical Data:

- Primary disease site, histology, molecular subtype/biomarkers
- SBFC state at each collection node
- Treatment delivered (modality, agent, dose/fractionation, dates)
- Rationale for sequencing decision (documented as free text + structured field)
- Response assessment (RECIST v1.1 / PERCIST as applicable)
- PFS1, PFS2, subsequent lines of therapy, OS

Dosimetric Data:

- EBRT: Total dose, fraction size, technique (SBRT/SABR vs. conventional), target volume, OAR constraints
- TRT: Radionuclide, administered activity, dosimetric method (where available), cumulative organ doses
- Cumulative BED calculations (where co-planning data are available) per the framework in **Supplementary File 3**

Toxicity Data:

- Adverse events graded per CTCAE v5.0 at each collection node
- Absolute lymphocyte count (ALC) documented at baseline, post-radiation, and at each interval follow-up

- Severe radiation-induced lymphopenia (ALC < 500/μL): date of onset, duration, clinical management

Quality of Life Data:

- Validated instrument: EORTC QLQ-C30 (with disease-specific module where applicable) administered at enrollment, every 24 weeks, and at end of active treatment
- Patient-Reported Outcome Measures (PROMs) archived as longitudinal time series per patient

Institutional Data:

- Participating site identifier (anonymized)
- MDT structure and composition at the time of treatment decision
- Availability of molecular imaging (PSMA-PET, FDG-PET, SPECT/CT)
- Availability of co-planning capability (radiation oncology + nuclear medicine physics)

Section 8: Endpoints

8.1 Primary Endpoints

Endpoint	Definition
PFS1	Time from enrollment to first documented radiographic or clinical progression
OS	Time from enrollment to death from any cause

Comparison: IRBT Cohort vs. Comparison Cohort (concurrent, same-institution controls).

8.2 Secondary Endpoints

Endpoint	Definition
PFS2	Time from enrollment to second documented progression or death
Duration of Systemic Therapy Continuation	Time from enrollment to permanent discontinuation of systemic therapy
Grade ≥3 Adverse Event Rate	Proportion of patients experiencing at least one Grade ≥3 CTCAE event during active treatment
Severe Lymphopenia Rate	Proportion of patients experiencing ALC < 500/μL, stratified by radiation modality and SBFC state

QoL Trajectory	Change from baseline EORTC QLQ-C30 global health score at 24 weeks and 12 months
SBFC Transition Frequency	Mean number of SBFC state transitions per patient per year
Inter-Rater SBFC Agreement	Kappa statistic for SBFC state agreement between independent blinded assessors (substudy)

Section 9: Statistical Considerations

9.1 Primary Analysis

Descriptive statistics will characterize the IRBT and Comparison Cohorts at baseline. Kaplan-Meier survival analysis will estimate PFS1 and OS in each cohort; log-rank testing will compare survival curves. Multivariable Cox proportional hazards regression will adjust for prespecified confounders: age, ECOG performance status, primary histology, number of prior lines of therapy, and SBFC state at enrollment.

9.2 Sample Size Rationale

The pilot target of 300 IRBT Cohort patients is powered for descriptive characterization and preliminary efficacy signal detection, not definitive superiority testing. This sample size will provide 80% power to detect a hazard ratio of 0.70 for PFS1 (median PFS 12 vs. 17 months), assuming 20% loss to follow-up over 3 years, at $\alpha = 0.05$ two-sided. Formal sample size re-estimation will occur at the 18-month interim analysis.

9.3 Handling of Missing Data

Multiple imputation by chained equations (MICE) will be used for missing covariates. Sensitivity analyses with complete-case and available-case datasets will be prespecified. Missing QoL data will be analyzed using mixed-effects models for repeated measures (MMRM).

9.4 Interim Analyses

A formal interim analysis for feasibility (enrollment rate, data completeness, SBFC assignment reliability) will be conducted at 18 months post-enrollment opening. No formal stopping rules for

efficacy or harm are stipulated at the registry stage; however, the Steering Committee retains authority to recommend registry suspension if unanticipated safety signals emerge.

Section 10: Governance Structure

10.1 Steering Committee

An international, multi-disciplinary Steering Committee will provide scientific oversight of the registry. Composition will include: radiation oncologists (minimum 2), medical oncologists (minimum 2), nuclear medicine physicians (minimum 1), medical physicists (minimum 1), biostatisticians (minimum 1), and patient advocates (minimum 1). At least three continents should be represented.

10.2 Data Safety and Monitoring

A Data Safety Monitoring Board (DSMB) independent of the Steering Committee will conduct annual safety reviews, with authority to recommend protocol modification or registry suspension based on pre-specified safety thresholds (e.g., Grade 5 event rate exceeding 5% attributable to protocol-directed sequencing).

10.3 Data Management and Quality

A central Electronic Data Capture (EDC) platform will be used. Source data verification will be conducted at all sites annually. Data quality metrics (completeness, accuracy, timeliness) will be reported to the Steering Committee quarterly.

10.4 Publication Policy

Results will be reported in accordance with STROBE reporting guidelines for observational studies. All participating institutions will be acknowledged. Authorship of primary registry publications will follow ICMJE criteria.

Section 11: Ethical Considerations

11.1 Regulatory Pathway

This registry requires Institutional Review Board (IRB) or Ethics Committee approval at each participating site. A coordinating site IRB will serve as the primary ethics authority for multi-site review where permitted by national regulations. International sites will comply with applicable national regulatory frameworks (e.g., NHS REC in the United Kingdom; CPP in France; MHLW in Japan).

11.2 Informed Consent

Written informed consent will be obtained from all participants prior to enrollment. A separate consent form will be used for the optional PROMs substudy and for any biospecimen collection associated with exploratory substudies.

11.3 Data Privacy and Security

All patient data will be de-identified at the point of entry using a coded participant identifier. Data transfers between sites and the central EDC will be encrypted. The registry will comply with GDPR (European Union), HIPAA (United States), and applicable data protection laws at all participating sites.

Section 12: Funding and Logistical Considerations

12.1 Pilot Phase Approach

The registry is proposed as a pilot initiative to establish proof-of-concept for a decentralized, SBFC-structured observational data infrastructure. The pilot phase (**at least 10 sites, 300 patients**) is designed to be achievable within the resource envelope of institutional investigator-initiated research programs and may not require dedicated external grant funding at launch.

12.2 Potential Funding Sources

Potential funding mechanisms for the full registry include:

- National Cancer Institute (NCI) R01/U01 mechanisms (United States)
- National Institute for Health and Care Research (NIHR) infrastructure funding (United Kingdom)
- European Organisation for Research and Treatment of Cancer (EORTC) collaborative mechanisms
- Industry partnerships with pharmaceutical or device manufacturers with a stake in theranostics or SBRT (subject to conflict-of-interest management policy)
- Philanthropic and foundation funding from cancer-focused organizations

12.3 Logistical Prerequisites for Participating Sites

Requirement	Minimum Standard
Radiation oncology capability	Linac with SBRT/**SABR** delivery capability (≥6 MV)
Molecular imaging	Access to PET/CT or SPECT/CT for response assessment
MDT infrastructure	Formalized tumor board meeting with radiation oncology and medical oncology participation
Data infrastructure	Ability to enter data into central EDC platform
Physics support	Medical physics support for cumulative dosimetric documentation
IRB/Ethics	Active ethics approval and regulatory authorization

12.4 Timeline for Registry Activation

Milestone	Projected Timeframe
Protocol finalization and IRB submission (pilot site)	Year 1, Q1–Q2
Coordinating site IRB approval	Year 1, Q3
EDC platform build and testing	Year 1, Q3–Q4
Enrollment opening (pilot site)	Year 1, Q4
Site activation (≥5 additional sites)	Year 2, Q1–Q2
18-month interim feasibility analysis	Year 3, Q2
Full registry expansion (≥10 sites)	Year 3, Q3

Abbreviations Used in This Document

Abbreviation	Definition
ALC	Absolute Lymphocyte Count

BED	Biologically Effective Dose
CTCAE	Common Terminology Criteria for Adverse Events
DSMB	Data Safety Monitoring Board
EBRT	External-Beam Radiotherapy
EDC	Electronic Data Capture
EORTC	European Organisation for Research and Treatment of Cancer
G-IRBT	Global Integrated Radiobiotherapy & Radiobiomedicine
GDPR	General Data Protection Regulation
HIPAA	Health Insurance Portability and Accountability Act
IRBT	Integrated Radiobiotherapy
IRB	Institutional Review Board
MDT	Multidisciplinary Team
MICE	Multiple Imputation by Chained Equations
MMRM	Mixed-Effects Model for Repeated Measures
OAR	Organs at Risk
OS	Overall Survival
PFS	Progression-Free Survival
PET/CT	Positron Emission Tomography / Computed Tomography
PROM	Patient-Reported Outcome Measure
QoL	Quality of Life
RECIST	Response Evaluation Criteria in Solid Tumors
SBFC	State-Based Framework Categorization
SBRT/SABR	Stereotactic Body Radiation Therapy / Stereotactic Ablative Radiotherapy
SPECT/CT	Single-Photon Emission Computed Tomography / Computed Tomography
STROBE	Strengthening the Reporting of Observational Studies in Epidemiology
TRT	Targeted Radionuclide Therapy

Note: This document is a proposed draft template for scientific and regulatory discussion. It is not a final registered protocol. All sections are subject to revision by the authors and any future investigative team adopting this framework for a formal registry submission.
